# Genetic and biochemical strategies for regulation of L-ascorbic acid biosynthesis in plants through the L-galactose pathway

**DOI:** 10.3389/fpls.2023.1099829

**Published:** 2023-03-15

**Authors:** Juan C. Castro, Carlos G. Castro, Marianela Cobos

**Affiliations:** ^1^ Unidad Especializada del Laboratorio de Investigación en Biotecnología (UELIB), Centro de Investigaciones de Recursos Naturales de la UNAP (CIRNA), Universidad Nacional de la Amazonia Peruana (UNAP), Iquitos, Peru; ^2^ Departamento Académico de Ciencias Biomédicas y Biotecnología (DACBB), Facultad de Ciencias Biológicas (FCB), Universidad Nacional de la Amazonia Peruana (UNAP), Iquitos, Peru

**Keywords:** ascorbate biosynthesis, regulation, genetic control, metabolic pathways, vitamin C

## Abstract

Vitamin C (L-ascorbic acid, AsA) is an essential compound with pleiotropic functions in many organisms. Since its isolation in the last century, AsA has attracted the attention of the scientific community, allowing the discovery of the L-galactose pathway, which is the main pathway for AsA biosynthesis in plants. Thus, the aim of this review is to analyze the genetic and biochemical strategies employed by plant cells for regulating AsA biosynthesis through the L-galactose pathway. In this pathway, participates eight enzymes encoded by the genes *PMI*, *PMM*, *GMP*, *GME*, *GGP*, *GPP*, *GDH*, and *GLDH*. All these genes and their encoded enzymes have been well characterized, demonstrating their participation in AsA biosynthesis. Also, have described some genetic and biochemical strategies that allow its regulation. The genetic strategy includes regulation at transcriptional and post-transcriptional levels. In the first one, it was demonstrated that the expression levels of the genes correlate directly with AsA content in the tissues/organs of the plants. Also, it was proved that these genes are light-induced because they have light-responsive promoter motifs (e.g., ATC, I-box, GT1 motif, etc.). In addition, were identified some transcription factors that function as activators (e.g., SlICE1, AtERF98, SlHZ24, etc.) or inactivators (e.g., SlL1L4, ABI4, SlNYYA10) regulate the transcription of these genes. In the second one, it was proved that some genes have alternative splicing events and could be a mechanism to control AsA biosynthesis. Also, it was demonstrated that a conserved cis-acting upstream open reading frame (5’-uORF) located in the 5’-untranslated region of the *GGP* gene induces its post-transcriptional repression. Among the biochemical strategies discovered is the control of the enzyme levels (usually by decreasing their quantities), control of the enzyme catalytic activity (by increasing or decreasing its activity), feedback inhibition of some enzymes (GME and GGP), subcellular compartmentation of AsA, the metabolon assembly of the enzymes, and control of AsA biosynthesis by electron flow. Together, the construction of this basic knowledge has been establishing the foundations for generating genetically improved varieties of fruits and vegetables enriched with AsA, commonly used in animal and human feed.

## 1 Introduction

Investigations about biological oxidations and related topics in the first half of the twentieth century have permitted to discover the vitamin C. Among these pioneering works are considered the investigations conducted by Albert Szent-Giörgyi and other scientists (Szent-Györgyi, 1928; King and Waugh, 1932; Hirst and Zilva, 1933; Hirst et al., 1933; Svirbely and Szent-Györgyi, 1933; Szent-Györgyi and Haworth, 1933; Szent-Gyoergyi, 1963), that together permitted to isolate and determine the chemical nature of the antiscorbutic factor, vitamin C. This compound was renamed L-ascorbic acid, which means “against scurvy”. When the molecular structure of AsA was elucidated, it was possible to develop chemical synthesis methods for its *in vitro* production (Reichstein et al., 1933; Haworth and Hirst, 1934; Haworth et al., 1934; Reichstein and Grüssner, 1934). Thus, synthetic AsA was quickly and cheaply available.

Of the multiple chemical approaches to AsA synthesis, the Reichstein–Grüssner process (Reichstein et al., 1933; Pappenberger and Hohmann, 2014) was the best. For this reason, it was used until the late 1990s as the major industrial process for AsA production to supply its great and growing demand. However, the Reichstein–Grüssner process provokes environmental issues because it employs multiple highly polluting chemicals for AsA synthesis. Consequently, the scientific community has been exploring alternative and innovative approaches. For example, the application of hybrid systems, which combine the classical Reichstein–Grüssner process and microbial cell platforms to produce AsA with more efficient and eco-friendly approaches (Ma et al., 2011; Wang et al., 2016; Wang et al., 2022). However, to date does not exist a method that meets the minimum requirements such as lower cost of production, efficiency, and effectiveness, eco-friendly, and high production capacity of production to satisfy the growing nutritional demand for this vitamin.

Another interesting possibility to supply the nutritional demand of AsA is fortifying some common foods of plant origin (e.g., fruits, tubers, etc.). This goal could be achieved by engineering the involved metabolic pathways through genetic improvement by targeting some specific genes encoding enzymes (Liu Y. et al., 2015; Pouvreau et al., 2018). A good metabolic pathway as a target for engineering could be the L-galactose pathway. This metabolic pathway is considered the main biosynthetic process to produce AsA in plants and to date is the best characterized (Wheeler et al., 1998; Valpuesta and Botella, 2004; Bulley and Laing, 2016; Fenech et al., 2019). Thus, we could generate novel plant varieties overproducing-AsA by using innovative approaches based on genome edition and synthetic biology methods (Feng et al., 2013; Belhaj et al., 2015).

However, to employ these novelty genetic modification approaches to generate plant varieties overproducing-AsA, it will be necessary to generate basic scientific knowledge. This comprises the complete and in-depth characterization of genes, enzymes, proteins, and metabolic pathways that control the biosynthesis, degradation, distribution, and accumulation of AsA in plant tissues and organs. In other words, it is indispensable to know the genetic and biochemical mechanisms that use the plants to regulate the AsA pool size. However, to achieve an in-depth understanding of these key processes, it is fundamental to have multi-omics resources (e.g., genomic, transcriptomic, proteomic, metabolomic, etc.) for each interesting plant species. Until now, multi-omics resources have been obtained for some plant species such as *Actinidia arguta* “kiwifruit” (Lei et al., 2022), *Capsicum annuum* “sweet pepper” (Alós et al., 2013), *Malpighia emarginata* “acerola” (Xu et al., 2022), *Myrciaria dubia* “camu-camu” (Castro et al., 2015b), and other plant species (Li et al., 2015; Li et al., 2017; Deng et al., 2022). In summary, with the accelerated increase and accessibility of multi-omics resources from diverse plant species, together with the decodification of the genetic and biochemical mechanisms controlling the metabolism and accumulation of AsA in plants, we will have a sufficient scientific basis to rationally develop plant varieties overproducing-AsA.

The development of plant varieties overproducing-AsA is more interesting because these foods derived from plants have a value-added. Because plants biosynthesize and store a myriad of nutritive and bioactive compounds such as polyphenols, pigments, and vitamins, among other compounds (Donado-Pestana et al., 2018; Castro et al., 2019). However, it is necessary to consider that overproducing-AsA mutants of *Solanum lycopersicum* “tomato” has disrupted some normal developmental processes. It was reported that mutant lines of tomato that produce high AsA content have impaired floral organ architecture. This impairment is specifically in the development of anthers and pollens, thus resulting in male sterility and producing parthenocarpic (seedless) fruits or having unviable seeds (Bulley et al., 2012; Deslous et al., 2021). Based on these results, it was hypothesized that seed development could be directly inhibited by the pro-oxidant activity of AsA, or AsA could regulate processes such as pollen viability, pollination, fertilization, ovule development, or embryo development (Bulley et al., 2012). Thus, when we are going to develop plant varieties overproducing-AsA, it will be fundamental to verify that key molecular and biochemical processes are unaffected to ensure its viability and appropriate use.

This review focuses on the genetic and biochemical strategies used by plant cells for regulating AsA biosynthesis through the L-galactose pathway based on scientific knowledge of the last twenty-five years.

## 2 Biosynthetic pathways of AsA in plants

**Figure 1:**
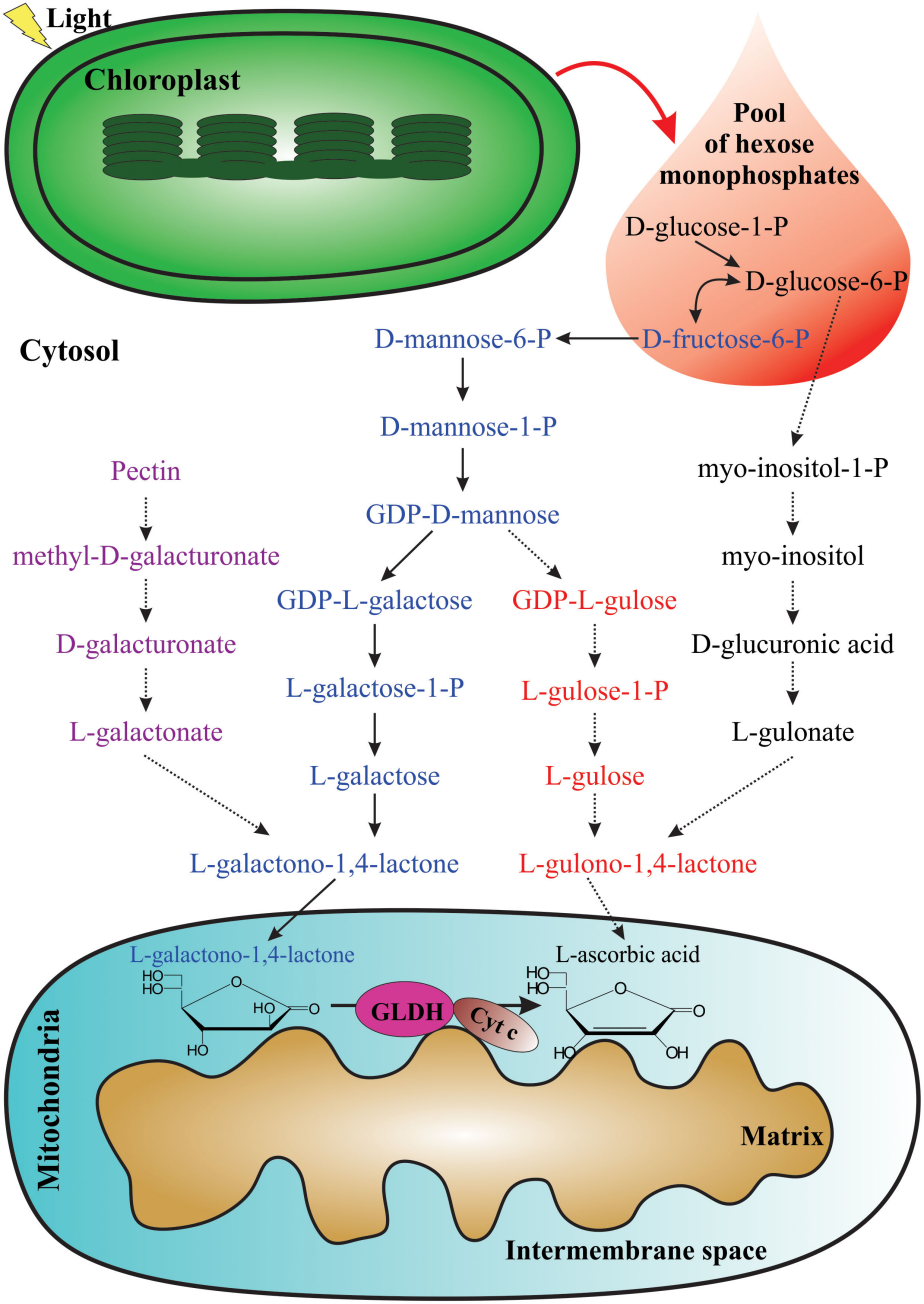
Proposed pathways for AsA biosynthesis in plants. The photosynthetic process provides the pool of hexose monophosphates as precursors for some AsA biosynthetic pathways. From left to right: D-galacturonic acid pathway (violet), L-galactose pathway (blue), L-gulose pathway (red), and myo-inositol pathway (black). The last enzymatic reaction for AsA biosynthesis occurs in the mitochondria.

**Figure 2:**
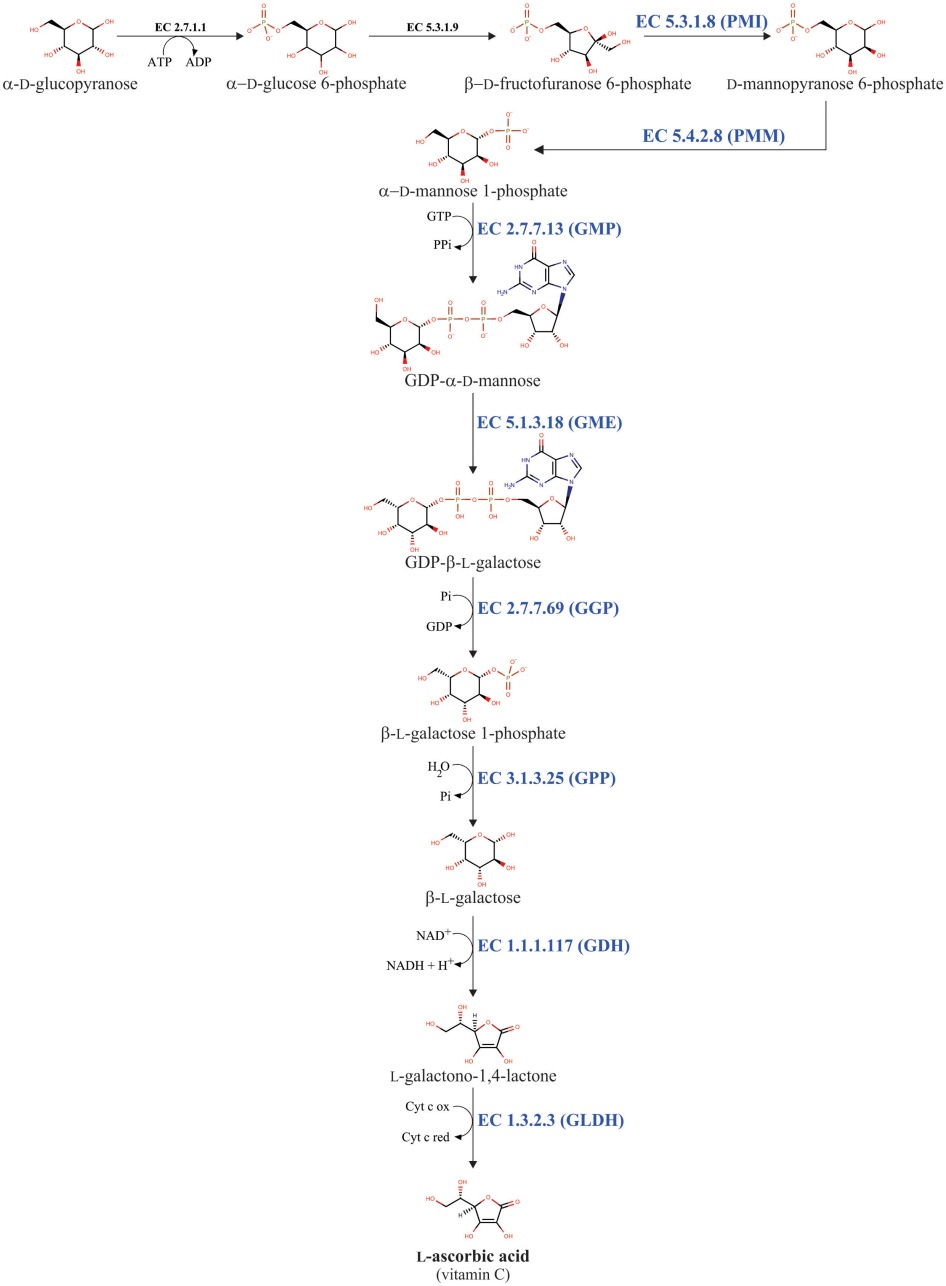
L-galactose pathway for AsA biosynthesis in plants. The eight enzymes involved in AsA biosynthesis are phosphomannose isomerase (PMI: EC 5.3.1.8), phosphomannomutase (PMM: EC 5.4.2.8), GDP-D-mannose pyrophosphorylase (GMP: EC 2.7.7.13), GDP-D-mannose 3’,5’-epimerase (GME: EC 5.1.3.18), GDP-L-galactose phosphorylase (GGP: EC 2.7.7.69), L-galactose-1-phosphate phosphatase (GPP: EC 3.1.3.25), L-galactose dehydrogenase (GDH: EC 1.1.1.117), and L-galactono-1,4-lactone dehydrogenase (GLDH: EC 1.3.2.3).

Multiple biosynthetic pathways of AsA have been suggested to be active in plants (**Figure 1**). The first one proposed was the L-galactose pathway (Wheeler et al., 1998), followed by the D-galacturonic acid pathway (Agius et al., 2003) and the L-gulose pathway (Wolucka and Van Montagu, 2003), and the last one suggested was the myo-inositol pathway (Lorence et al., 2004). Of these metabolic routes, the L-galactose pathway for AsA biosynthesis, which converts D-fructose 6-phosphate into AsA (**Figure 2**), is best supported by multiple genetic and biochemical studies. From this biosynthetic pathway, the eight genes encoding enzymes were identified and cloned, and the catalytic activities of the corresponding enzymes have been functionally well-characterized in higher plants. These include phosphomannose isomerase (PMI: EC 5.3.1.8) (Maruta et al., 2008), phosphomannomutase (PMM: EC 5.4.2.8) (Qian et al., 2007), GDP-D-mannose pyrophosphorylase (GMP: EC 2.7.7.22) (Conklin et al., 1999), the GDP-D-mannose 3’,5’-epimerase (GME: EC 5.1.3.18) (Wolucka and Van Montagu, 2003), the GDP-L-galactose phosphorylase (GGP: EC 2.7.7.69) (Laing et al., 2007), L-galactose-1-phosphate phosphatase (GPP: EC 3.1.3.25) (Laing et al., 2004a), L-galactose dehydrogenase (GDH: EC 1.1.1.117) (Wheeler et al., 1998; Gatzek et al., 2002; Laing et al., 2004b), and L-galactono-1,4-lactone dehydrogenase (GLDH: EC 1.3.2.3) (Mapson and Breslow, 1958; Oba et al., 1995; Ostergaard et al., 1997).

Until now, only are available three-dimensional (3D) structures of some enzymes from the L-galactose pathway. These 3D structures have been obtained using X-ray crystallography analysis. These include the GME (Major et al., 2005) and GMP enzymes (Zhang et al., 2022a) from *Arabidopsis thaliana* “thale cress”, the GDH enzyme from *Spinacia oleracea* “spinach” (Vargas et al., 2022), and the GLDH enzyme from *Brassica oleracea* “cauliflower” (Soufari et al., 2020) and *Myrciaria dubia* “camu-camu” (PDB code: 7SML, unpublished results). To date, our research team has been crystallizing the GME and GDH enzymes from *Myrciaria dubia* “camu-camu”, and shortly will be determined the three-dimensional structures of both enzymes. Camu-camu is an AsA hyper-producer plant native to the Amazon basin region (Rodrigues et al., 2001; Castro et al., 2018).

The L-galactose pathway is not an exclusive pathway for AsA biosynthesis. Because the L-galactose pathway generates the activated sugar nucleotide GDP-D-mannose (**Figure 2**). This compound is a metabolic precursor to produce, through a nucleotide interconversion pathway, other guanosine-containing sugar nucleotides such as GDP-L-fucose, GDP-D-rhamnose, and GDP-L-galactose (Barber, 1968; Baydoun and Fry, 1988; Wheeler et al., 1998; Wolf-Dieter, 1998). GDP-L-galactose is further used as a metabolic intermediate for both AsA biosynthesis and cell wall polysaccharides biosynthesis (Baydoun and Fry, 1988; Wheeler et al., 1998), whereas the other GDP-containing sugar nucleotides (i.e., GDP-D-mannose, GDP-L-fucose, and GDP-D-rhamnose) are exclusively needed to biosynthesize structural polysaccharides in the cell walls (e.g., galactomannans, glucomannans, rhamnogalacturonan II, etc.) of plants (Baydoun and Fry, 1988; Voxeur et al., 2011). Finally, these GDP-containing sugar nucleotides are essential to make post-translational modifications, including protein N-glycosylation and glycosylphosphatidylinositol anchoring (Lerouge et al., 1998; Strasser, 2016). In summary, the L-galactose pathway can be divided into two biosynthetic steps. The first step includes the consecutive biochemical reactions catalyzed by the enzymes PMI, PMM, GMP, and GME, which convert β-D-fructofuranose 6-phosphate into GDP-β-L-galactose. This first step provides the metabolic intermediaries GDP-D-mannose and GDP-β-L-galactose, which are required to biosynthesize cell wall components and for the post-translational modifications described. The second one includes biochemical reactions catalyzed by the enzymes GGP, GPP, GDH, and GLDH, which together transform GDP-β-L-galactose into AsA. This second step is exclusively dedicated to AsA biosynthesis, for this reason, the reaction catalyzed by the GGP enzyme is considered the committed step for AsA biosynthesis in plants (Wheeler et al., 1998; Laing et al., 2007).

As in plants, AsA has essential and pleiotropic functions in all kinds of cells, tissues, and organs (photosynthetic and non-photosynthetic ones), it is fundamental to ensure AsA supplying by *in situ* biosynthesis and/or long-distance transport (of AsA or some of its metabolic intermediaries) from their biosynthesis sites. In some plant species, such as camu-camu was demonstrated that several genes of the L-galactose pathway (*GMP*, *GME*, *GGP*, *GPP*, *GDH*, and *GLDH*) and their corresponding encoded enzymes are expressed and are catalytically active, respectively, in leaves and fruits (fruit pulp and fruit peel). These results suggest that these tissues of camu-camu are capable of biosynthesizing AsA *in situ* (Castro et al., 2015a), and probably the high accumulation of AsA in their fruits is due to the occurrence of both processes, *in situ* biosynthesis and long-distance transport from organs with the highest biosynthetic activity of AsA, such as leaves. Also, in *Medicago sativa* and thale cress was demonstrated a long-distance transport of AsA by the phloem from source leaves to sink tissues such as developing shoot tips, root tips, developing inflorescences, very young flower buds, siliques, and flowers (Franceschi and Tarlyn, 2002). In *Solanum tuberosum* “potato” it was hypothesized that AsA accumulation in tubers depends principally on long-distance transport. This hypothesis was supported due to the high correlation between changes in the AsA content of source leaves, AsA content of phloem, and AsA accumulation in developing tubers (Tedone et al., 2004). Additionally, it was suggested that biosynthesis *in situ* supplies AsA during tubers development (tuberization). Because during tuberization the complete set of genes encoding enzymes of the L-galactose pathway (i.e., *PMI*, *PMM*, *GMP1*, *GMP2*, *GME1-3*, *GGP1*, *GGP2*, *GPP*, *GDH*, and *GLDH*) increases significantly its expression levels, correlating with developmental stage and AsA content in tubers (Blauer et al., 2013). Together these results suggest that AsA accumulation in non-photosynthetic organs (e.g., fruits, tubers, flowers, etc.) occurs by both *in situ* biosynthesis and long-distance transport from source leaves but will be necessary to quantify the contribution of each process into AsA accumulation in non-photosynthetic organs.

## 3 Enzymes of the L-galactose pathway for AsA biosynthesis

### 3.1 Phosphomannose isomerase (PMI: EC 5.3.1.8)

Phosphomannose isomerase is encoded by two *PMI* genes of thale cress (Maruta et al., 2008). The *AtPMI1* gene (*At3g02570*) contains five exons and is located on chromosome 3 (https://www.ncbi.nlm.nih.gov/gene/820656), whereas the *AtPMI2* gene (*At1g67070*) contains five exons and is located on chromosome 1 (https://www.ncbi.nlm.nih.gov/gene/843027). The *AtPMI1* gene and its corresponding enzyme are constitutively expressed in vegetative (i.e., root, stem, leaf, and cauline leaf) and reproductive organs (inflorescence) under normal growth conditions, whereas the *AtPMI2* gene and its encoded enzyme do not express in any organs under illumination (Maruta et al., 2008). Also, it was recorded that when the plants are exposed to continuous illumination, the expression of the *AtPMI1* gene is induced significantly in the leaves, correlating with an increase in their AsA content. In contrast, when the plants are under long-term darkness, the expression of the *AtPMI2* gene is induced in the leaves, but with a decrease in their AsA content. Additionally, it was recorded that the *PMI1* gene presents a diurnal expression pattern that matches the total catalytic activity of the PMI enzymes and the total AsA content in leaves. Finally, it was proved that a reduction in the *AtPMI1* gene expression, using an RNA interference approach to knockdown the *PMI1* gene, results in a significant decrease in the *AtPMI1* mRNA level, in the *At*PMI1 protein content and in the catalytic activity of the *At*PMI1 enzyme, which correlated with a decreased AsA content (from 47 to 65% lower content than control plants) in leaves. However, when the *AtPMI2* gene is knocking-out, thus blocking its expression completely, it does not affect the total AsA content in leaves of the knock-out *AtPMI2* gene plants (Maruta et al., 2008). In summary, these results provide genetic and biochemical evidence that the encoded enzyme of the *AtPMI1*gene, but not of the *AtPMI2* gene, is involved in the biosynthesis of AsA in thale cress plants.

These genes encode the *At*PMI1 and *At*PMI2 enzymes, with 432 and 441 amino acid residues, respectively (Maruta et al., 2008). Both enzymes are a zinc-dependent monofunctional aldose-ketose isomerase of type I that possesses the conserved cupin domain (Dunwell, 1998; Maruta et al., 2008). The *At*PMI1 has a good sequence identity (41%) with a reported PMI enzyme of type I from the fungus *Candida albicans* (*Ca*PMI) (Maruta et al., 2008). PMI is a metal-dependent aldose-ketose isomerase that catalyzes the reversible isomerization of D-fructose 6-phosphate and D-mannose 6-phosphate in prokaryotic and eukaryotic organisms (Roux et al., 2011). The reaction catalyzed by PMI is the first catalytic step in conducting hexose monophosphates into D-mannose 6-phosphate. The PMI enzymes have been classified into four types on the basis of their amino acid sequence identity, physicochemical characteristics, and kinetic properties (Proudfoot et al., 1994; Jensen and Reeves, 1998; Roux et al., 2011). PMIs of type I are present in some prokaryotic organisms (e.g., *Escherichia coli*, *Salmonella typhimurium*) and all eukaryotic organisms (e.g., yeasts, animals, plants, etc.). However, PMIs of types II, III, and IV are only restricted to prokaryotic organisms, commonly gram-negative bacteria such as *Acinetobacter calcoaceticus*, *Pseudomonas aeruginosa*, *Rhizobium meliloti, Rhodospirillium rubrum*, *Xanthomonas campestris*, etc. (Proudfoot et al., 1994; Jensen and Reeves, 1998).

Structurally, the PMI enzyme belongs to the cupin superfamily of proteins (Dunwell, 1998). These proteins have a putative β-barrel shape (“cupa” is the Latin term for small barrel). Typically, the cupin domain comprises two conserved motifs containing two β strands. The first motif has the consensus sequence G(x)_5_HxH(x)_3,4_E(x)_6_G, while the second one has the consensus sequence G(x)_5_PxG(x)_2_H(x)_3_N. These two conserved motifs are linked by a less conserved intermotif sequence. The intermotif sequence has variable lengths (from 15 to > 50 amino acid residues) and contains two β strands (Dunwell et al., 2001).To date has been determined the Three-dimensional (3D) structure of the *Ca*PMI enzyme (Cleasby et al., 1996; Ahmad et al., 2018). The *Ca*PMI enzyme shows three domains: two similar antiparallel β-strand domains and a helical domain. The catalytic domain (residues 11–52, 266–332) is flanked by a helical domain and a carboxy-terminal β-jelly roll domain. The catalytic domain contains the active site, which is a profound and open cavity of suitable dimensions to hold D-fructose 6-phosphate or D-mannose 6-phosphate. The deepest part of the active site contains a single Zn^2+^ ion hexacoordinated by three oxygen and three nitrogen ligands provided by the sidechains of Gln111, His113, Glu138, and His285. The zinc cofactor has a structural and catalytic role in the reaction catalyzed by the *Ca*PMI enzyme (Cleasby et al., 1996; Ahmad et al., 2018).

The biochemical properties of the recombinant *At*PMI enzymes show some particularities. Both *At*PMI1 and *At*PMI2 enzymes lack a signal peptide, which indicates that these enzymes are located in the cytosol. The molecular weights of the enzymes are 48.5 kDa (*At*PMI1), and 49.2 kDa (*At*PMI2). The optimal pH for the *At*PMI1 and *At*PMI2 enzymes is 7.5, and the optimal temperatures for both enzymes are 52 and 48°C, respectively. The *At*PMI1 and *At*PMI2 enzymes follow the kinetics of Michaelis-Menten. The *K*_M_ values for D-mannose 6-phosphate of the *At*PMI1 and *At*PMI2 enzymes are 41.3 ± 4.2 μM and 372 ± 13 μM, respectively. The *V*_max_ values for D-mannose 6-phosphate of the *At*PMI1 and *At*PMI2 enzymes are 1.89 and 22.5 μmol.min^-1^.mg protein^-1^, respectively. Both enzymes are competitively inhibited by Zn^2+^, Cd^2+^, and AsA. For the *At*PMI1 enzyme, the *K*_i_ values of the three inhibitors are 32.0, 11.0, and 1,100 μM, respectively. However, for *At*PMI2, the *K*_i_ values of the three inhibitors are 2.1, 7.8, and 1,400 μM, respectively. The inhibitory effect of AsA on the *At*PMI1 and *At*PMI2 enzymes suggests that ascorbate is a feedback inhibitor for these enzymes, taking into account that the cytosolic concentration of AsA is more than 21 mM (Zechmann, 2011).

### 3.2 Phosphomannomutase (PMM: EC 5.4.2.8)

Phosphomannomutase is encoded by the *PMM* gene. This gene has a similar structural organization (i.e., number and distribution of exons and introns) but distinct chromosomal localization and gene copy number in monocotyledons and dicotyledons (Yu et al., 2010). Thus, the *PMM* gene of thale cress (*At2g45790*) contains ten exons and is located on chromosome 2 (https://www.ncbi.nlm.nih.gov/gene/819187). In *Oryza sativa “*rice*”*, the *PMM* gene (*Os04g0682300*) contains eleven exons and is located on chromosome 4 (https://www.ncbi.nlm.nih.gov/gene/4337437), but in *Solanum lycopersicum* “tomato”, the *PMM* gene possesses twelve exons and is located on chromosome 5 (https://www.ncbi.nlm.nih.gov/gene/778245). Also, it is known that genomes of plant species like thale cress, tomato, rice, and *Brachypodium distachyon* contains a unique copy of the *PMM* gene. However, other plant species such as *Aegilops tauschii*, *Hordeum vulgare*, *Triticum urartu*, *Triticeae turgidum*, and *Triticum aestivum* harbor in their genomes from 2 to 6 copies of the PMM gene (Zou et al., 2006; Yu et al., 2010).

In some studied plant species was demonstrated that the gene encoding the PMM enzyme is involved in the biosynthesis of AsA. First, in thale cress and *Nicotiana benthamiana*, the *PMM* gene has constitutive transcriptional patterns in both vegetative (i.e., roots, stems, and leaves) and reproductive organs (i.e., flowers and immature fruits) (Qian et al., 2007). When the expression level of the *PMM* gene of *Nicotiana benthamiana* was lowered, using a pea early browning virus-mediated gene silencing approach, their leaves showed a significant decrease in AsA content. However, when the expression level of the *PMM* gene of the same plant species was increased using a viral-vector-mediated ectopic expression, their tissues had an increase in AsA content from 20 to 50%. Similarly, when a transgene encoding the *At*PMM–GFP fusion protein was expressed in thale cress, it was recorded a significant increase in AsA content from 25 to 33% (Qian et al., 2007). Additionally, it was demonstrated that in *Malpighia glabra*, the AsA contents in the ripening fruits and leaves correlate with the expression levels of the *MgPMM* gene. Furthermore, transgenic *Nicotiana tabacum* overexpressing the *MgPMM* gene showed a significant increase in AsA contents. The increase in AsA content correlates with the mRNA levels of the *MgPMM* gene and the catalytic activities of the *Mg*PMM enzyme (Badejo et al., 2009a). Finally, it was proved that in *Dendrobium officinale* the *DoPMM* gene is expressed in roots, stems, leaves, and flowers. To test the participation of the *DoPMM* gene in the biosynthesis of AsA and polysaccharides, the researchers have produced transgenic lines of *Arabidopsis thaliana* overexpressing the *DoPMM* gene. The three transgenic lines of *Arabidopsis thaliana* showed an increase in AsA accumulation (from 31 to 40%) and polysaccharide content (from 22 to 77%) (He et al., 2017).

The PMM enzyme catalyzes the reversible isomerization of D-mannose 6-phosphate into D-mannose 1-phosphate (Oesterhelt et al., 1997). This enzymatic reaction is achieved by the intramolecular transfer of the phosphoryl group through a phosphoenzyme intermediate (Rose, 1986; Martínez Cuesta et al., 2016). The PMM enzyme belongs to the glucose biphosphate family because it uses the cofactors D-glucose 1,6-biphosphate or D-mannose 1,6-biphosphate as phosphate donors (Guha and Rose, 1985; Rose, 1986). Also was demonstrated that the PMM enzymes from eukaryotic organisms (i.e., yeasts, mammals, and plants) share a well-conserved DXDX(T/V) motif at their amino-terminal region that functions as an intermediate acceptor of the phosphoryl moiety (Collet et al., 1998; Qian et al., 2007; Badejo et al., 2009a; Yu et al., 2010; He et al., 2017). In the conserved DXDX(T/V) motif, the first aspartate is the phosphorylated residue in the PMM phosphoenzyme (Collet et al., 1998).

Structurally, the PMM enzyme belongs to the haloalkanoic acid dehalogenase (HAD) superfamily (Burroughs et al., 2006). These enzymes catalyze nucleophilic substitution reactions at phosphorus or carbon centers. To make these reactions, the enzymes use a conserved aspartate carboxylate in covalent catalysis. The PMM enzyme, like most HAD superfamily proteins, consists of the cap and the core domains. These domains are opened to accommodate the substrate and then closed to provide a solvent-free environment for the catalysis (Silvaggi et al., 2006). The core catalytic domain of the HAD superfamily adopts the typical topology of the Rossmannoid class of α/β folds. In other words, HAD fold consist of a three-layered α/β sandwich composed of repeating β-α units. In addition, the HAD fold shows the squiggle and the flap motifs that play key roles in HAD superfamily catalysis (Burroughs et al., 2006). Also, comparisons of amino acid sequences show that all members of the HAD superfamily possess four highly conserved sequence motifs (Aravind et al., 1998). These conserved sequence motifs are spatially arranged around a single binding cleft at the carboxyl-terminal region of the strands of the central sheet that forms the active site of the HAD superfamily (Burroughs et al., 2006).

The biochemical properties of the recombinant PMM enzyme from *Arabidopsis thaliana* (*At*PMM) have been determined and show some properties. The *At*PMM enzyme has a molecular weight of 27.7 kDa. The enzyme requires D-glucose 1,6-bisphosphate as an enzymatic cofactor to convert D-mannose 1-phosphate into D-mannose 6-phosphate. The optimal pH and temperature for the *At*PMM enzyme are 7.5 and 30°C, respectively. The *At*PMM enzyme follows the kinetics of Michaelis-Menten. The *K*_M_ value for D-mannose 1-phosphate of the *At*PMM enzyme is 29.7 μM. The *V*_max_ value to convert D-mannose 1-phosphate into D-mannose 6-phosphate of the *At*PMM enzyme is 14.4 μmol.min^-1^.mg protein^-1^ (Qian et al., 2007).

### 3.3 GDP-D-mannose pyrophosphorylase (GMP: EC 2.7.7.13)

In a relatively short period of time after the discovery of the L-galactose pathway for AsA biosynthesis in plants (Wheeler et al., 1998), several of their genes encoding enzymes (including the *VTC1* gene) were identified in mutant plants isolated from a collection of AsA-deficient thale cress mutants. To obtain the collection of mutant plants, the seeds of thale cress Columbia (Col-0) wild-type ecotype were mutagenized with ethyl methanesulfonate (Conklin et al., 1996). From this collection, one of the first mutant plants to be isolated and thoroughly characterized was the AsA deficient (≈30% of the wild-type AsA levels) and ozone-hypersensitive thale cress mutant vitamin c-1 (*vtc1-1*, formerly known as *soz1*) (Conklin et al., 1996). The reduced accumulation of AsA and the ozone sensitivity of the thale cress mutant *vtc1-1* is conferred by a semi-dominant monogenic mutation in the *VTC1* gene, which maps to chromosome 2 of thale cress (Conklin et al., 1996). Also, according to the results of D-[U-^14^C]glucose labeling assays using detached leaves with petioles of five-week-old plants, the AsA deficiency in these mutant plants is due to a biosynthetic defect (Conklin et al., 1997). Furthermore, Smirnoff’s research team used a combination of biochemical, molecular, and genetic techniques to demonstrate that the *VTC1* gene encodes the enzyme GDP-D-mannose pyrophosphorylase (GMP enzyme) (Conklin et al., 1999). Now, it is known that the gene encoding the GMP enzyme of thale cress (*CYT1, At2g39770*) contains six exons and is located on chromosome 2 (https://www.ncbi.nlm.nih.gov/gene/818562).

The GMP enzyme catalyzes a reversible conversion of D-mannose 1-phosphate plus GTP into GDP-D-mannose plus pyrophosphate (Wheeler et al., 1998; Conklin et al., 1999). Consequently, the catalytic activity of the GMP enzyme supplies the metabolic intermediate GDP-D-mannose. This metabolic intermediate is a common substrate for multiple pathways, such as the biosynthesis of cell-wall carbohydrates, the glycosylation of proteins, and the biosynthesis of AsA (Conklin et al., 1999). Subsequently was demonstrated that the catalytic activity of the GMP enzyme is lower (≈35%) in leaf extracts of mutants than in wild-type plants. The decreased catalytic activity of the GMP enzyme of the thale cress mutant *vtc1-1* was associated with a point mutation (change of cytosine to thymine) at position +64 relative to the start codon. This missense mutation changes a highly conserved proline to serine at position 22 (P22S) in the amino acid sequence of the GMP enzyme. Finally, it was demonstrated that the point mutation does not affect the mRNA levels of the *VTC1* gene, thus was hypothesized that the point mutation could affect the enzyme activity or stability rather than transcription or mRNA stability (Conklin et al., 1999).

**Figure 3:**
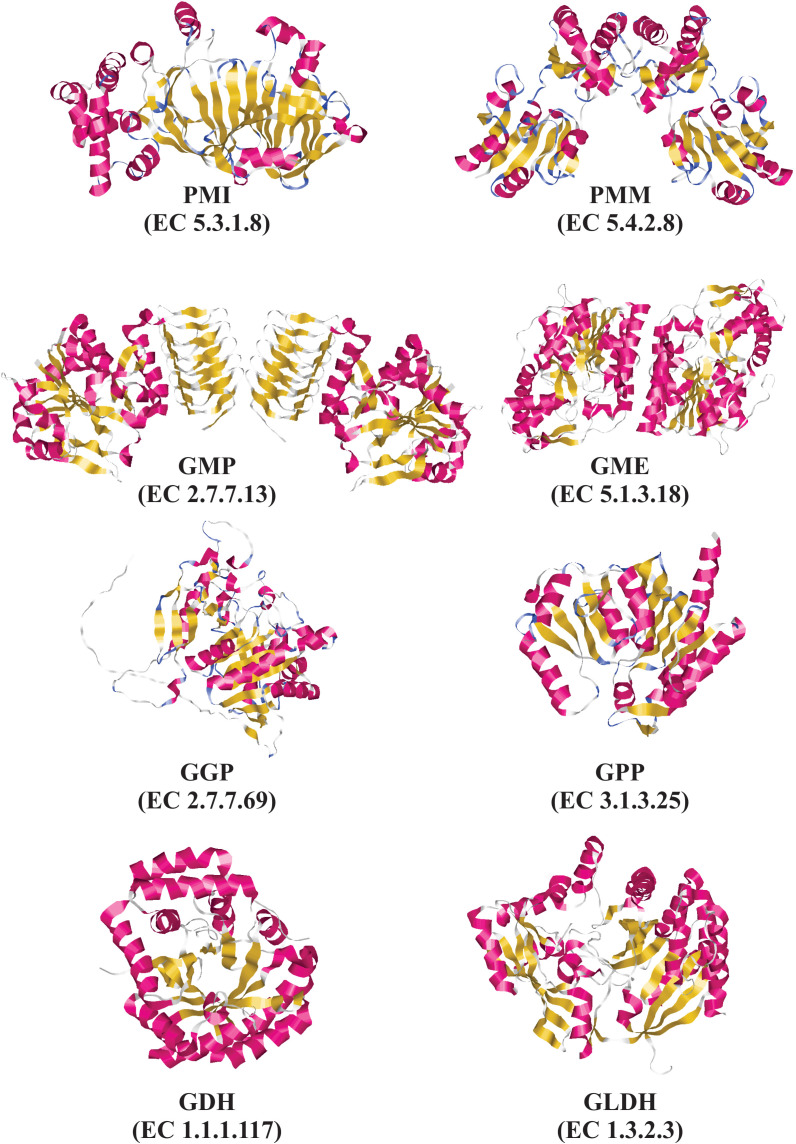
Experimental and predicted tridimensional structures of enzymes from the L-galactose pathway for AsA biosynthesis. Experimentally determined tridimensional structures by X-ray diffraction for the following enzymes: GMP from *Arabidopsis thaliana* “thale cress” (PDB code: 7X8K), GME from *Arabidopsis thaliana* “thale cress” (PDB code: 2C54), GDH from *Spinacia oleracea* “spinach” (PDB code: 7SMI), and GLDH from *Myrciaria dubia* “camu-camu” (PDB code: 7SML). Predicted tridimensional structures determined by using SWISS-MODEL for the following enzyme: PMI from *Arabidopsis thaliana* using as a template the three-dimensional structure of PMI from *Candida albicans* (PDB code 1PMI). Predicted tridimensional structures determined by using AlphaFold for the following enzymes: GGP from *Arabidopsis thaliana* (AlphaFoldDB: Q8RWE8), and GPP from *Arabidopsis thaliana* (AlphaFoldDB: Q9M8S8). Tridimensional structures were drawn using RasMol v2.7.5.

Structurally, the GMP enzyme from thale cress has a molecular weight of 41 kDa, and apparent molecular weights of ≈80 and 170 kDa (Zhao and Liu, 2016; Zhang et al., 2022b). Also, the GMP enzyme has an oligomer-forming monomer (protomer), and their oligomers vary in size and organization. For example, a dodecamer consists of a top hexamer and a basal hexamer. The top hexamer is formed by the trimerization of dimers, while the basal hexamer is like an appendix of the top hexamer because does not exist direct inter-dimer interaction within the basal hexamer (Zhang et al., 2022b). The GMP enzyme is composed of seven α-helices, seven η-helices, and 30 β-strands (**Figure 3**). These secondary structures are organized in two domains: an amino-terminal Rossmann fold-like domain (catalytic domain) that contains 14 helical elements and 13 β-strands (β1–β13) and a C-terminal left-handed β-helix (LβH) domain that consists of 17 β-strands (β14–β30) (Zhang et al., 2022b). The catalytic domain is built up of a β-sheet core flanked by α-helices and belongs to the glycosyltransferase (GT)-A fold (Coutinho et al., 2003; Lairson et al., 2008; Drula et al., 2022). The LβH domain is involved in the oligomerization and allosteric regulation of some enzymes, such as the ADP-glucose pyrophosphorylase (Figueroa et al., 2022).

To date, kinetic parameters for the GMP enzyme are missing, but the catalytic activity of this enzyme is detectable in leaves of thale cress (Conklin et al., 1999). Also, the GMP enzyme is active in leaves, fruit pulp, and fruit peel of *Myrciaria dubia* “camu-camu” (Castro et al., 2015a).

### 3.4 GDP-D-mannose 3’,5’-epimerase (GME: EC 5.1.3.18)

The first time to be isolated and cloned the gene encoding the GME enzyme of thale cress was in the early 2000s. The gene encoding the GME enzyme contains six exons, has a unique copy, and is located on chromosome 5 of the thale cress (Wolucka et al., 2001). Other plant species also possess a single copy of the *GME* gene, these include *Oryza sativa* “rice”(Watanabe et al., 2006), *Medicago sativa* “alfalfa”(Ma et al., 2014), and *Prunus persica* “peach” (Imai et al., 2009). However, *Solanum lycopersicum* “tomato” contains two homologous of the *GME* gen. The *SlGME1* gene is located on chromosome 1 (Stevens et al., 2007), whereas the *SlGME2* is located on chromosome 9 (Zou et al., 2006; Stevens et al., 2007). The nucleotide sequences of both genes have an identity of 79%, whereas the deduced amino acid sequences of *Sl*GME1 and *Sl*GME2 share a similarity of 92% (Zhang et al., 2011).

The participation of the encoded enzymes of both genes (*SlGME1* and *SlGME2*) in the metabolic pathways for AsA and cell wall biosynthesis of tomato was demonstrated by RNAi-silencing or over-expressing both genes. Thus, tomato lines with RNAi-silenced *SlGME* genes presented low levels of AsA (from 40 to 60%) compared to wild-type plants. Also, these tomato lines showed growth defects because key processes such as cell division and expansion were affected. Additionally, the RNAi-silenced tomato lines showed changes in the composition and structure of non-cellulosic cell-wall compounds (hemicelluloses and pectins), as well as a modification in the content of D-mannose and L-galactose in the cell wall, which depends on the catalytic activity of the GME enzymes (Gilbert et al., 2009). Additionally, it was reported that the GME enzymes encoded by both genes function in the biosynthesis of AsA and non-cellulosic cell wall polysaccharides. But, was registered a preferential expression of each *SlGME* gene in distinct tissues of tomato, suggesting a sub-functionalization and specialization of the *Sl*GME1 and *Sl*GME2 enzymes in the biosynthesis of cell wall components in specific tissues of tomato (Mounet-Gilbert et al., 2016). Finally, it was reported that transgenic tomato plants over-expressing the genes *SlGME1* and *SlGME2* presented a significant increase in total AsA in leaves and red fruits compared with wild-type plants. The researchers also demonstrated that the transgenic tomato plants over-expressing both genes enhanced stress tolerance when subjected to some stressful conditions, such as exposure of plants to oxidative stress with methyl viologen, cold stress, and salt stress (Zhang et al., 2011). Together these findings indicate that in plants, the GME enzymes are key players in AsA and non-cellulosic cell-wall polysaccharide biosynthesis.

Structurally, GME is a homodimeric enzyme that belongs to the short-chain dehydrogenase/reductase protein superfamily (Wolucka et al., 2001), which shares the extended Rossmann fold with a modified glycine-rich nucleotide-binding domain (GAGGFIA instead of GXGXXG) involved in NAD(P)-binding, a catalytic triad ([ST]xnYx3K) and similar mechanisms of catalysis (Persson and Kallberg, 2013; Da Costa et al., 2021). The tridimensional structure of the GME enzyme shows two domains (Major et al., 2005). The first one is the modified Rossmann fold, which binds the enzyme cofactor NAD^+^, and the second one is the substrate domain, which binds sugar nucleotide. The organization of the secondary structures varies in both domains. Thus, the modified Rossmann fold has in its β-sheet seven parallel β-strands (β1, β2, β3, β4, β5, β7, and β12) flanked by six alpha-helices (three on each face). Additionally, the modified Rossmann fold has added secondary structures after β4 and β5 strands, which participate in the substrate binding domain. The substrate binding domain is composed principally of alpha-helices with two short parallel β-sheets (β8 and β11 plus β6 and β15) and an antiparallel β-sheet (β10 and β13). Three loops from the carboxy-terminal fold up against the modified Rossmann fold, including two alpha helices (αK and αM) and amino acid residues from position 363 to 375. The homodimeric structure of the GME enzyme is thanks to the alpha-helices from one of the faces of the modified Rossmann fold forming the dimer interface (Major et al., 2005).

GME is an uncommon enzyme for several reasons. First, the GME enzyme performs three biochemical reactions that include oxidation, epimerization, and reduction. Second, the enzyme acts on three different carbohydrates, even preserving its substrate selectivity. Finally, the GME enzyme is capable of accomplishing two different epimerization reactions using the same acid/base catalytic dyad of amino acid residues (Major et al., 2005).

To date, GME is the enzyme for AsA biosynthesis that has been studied widely on its catalytic mechanism. The epimerase catalyzes at least two distinct epimerization reactions on the GDP-D-mannose substrate (Wolucka and Van Montagu, 2003), and releases, in addition to the well-known GDP-L-galactose, two additional products GDP-L-gulose and GDP-D-altrose in an equilibrium ratio of 72:20:4:4. This suggest that the GME enzyme does not differentiate between the C3’ and C5’ position as initial epimerization site (Gevaert et al., 2020). The reaction catalyzed for the GME enzyme proceeds by C4’ oxidation of the substrate GDP-α-D-mannose followed by epimerization of the C5’ position to generate GDP-β-L-4-keto-gulose. This intermediate is reduced to give GDP-β-L-gulose or epimerized in the C3’ position to produce GDP-β-L-4-keto-galactose, subsequently the C4’ position is reduced to generate GDP-β-L-galactose (Major et al., 2005). Both epimerization reactions are performed by the same catalytic dyad (cysteine and lysine), indicating that both amino acid residues are reactivated in each catalytic cycle of the GME enzyme (Gevaert et al., 2020).

The biochemical characteristics of the native GME enzyme of thale cress show some particularities. The GME enzyme has a sequence length of 377 amino acid residues, and it is the most conserved enzyme (≈90% identity) between dicotyledons and monocotyledons plants (Wolucka and Van Montagu, 2007). In thale cress, the denatured GME enzyme has a molecular weight of ≈43 kDa, but the native one is dimeric with a molecular weight of 84 kDa, composed of two apparently identical subunits (Wolucka et al., 2001). Regarding the kinetic parameters of the GME enzymes, the native one purified from thale cress and its two recombinant versions (amino-terminal His-tag and amino-terminal GST-tag) show some differences. Thus, the *K*_M_ values for GDP-D-mannose are 4.5, 18.0, and 31.0 μM, respectively. The *K*_cat_ values are 0.041, 0.007, and 0.010 s^-1^, respectively, and the *K*_cat_/*K*_M_ values are 9.1, 0.4, and 0.3 mM^-1^.s^-1^, respectively (Wolucka and Van Montagu, 2003). In addition, GDP (*K*_i_ = 0.7 μM) and GDP-D-glucose (*K*_i_ = 5 μM) are strong competitive inhibitors of the native epimerase. NAD^+^ and NADP are activators of the enzyme, increasing their activity at 145 and 110% compared with the control, respectively. However, the reduced forms of these coenzymes (NADH and NADPH) inhibit the enzyme, decreasing its catalytic activity at 78 and 88% compared with the control, respectively (Wolucka and Van Montagu, 2003).

### 3.5 GDP-L-galactose phosphorylase (GGP: EC 2.7.7.69)

GDP-L-galactose phosphorylase is encoded by the *VTC2* gene (*At4g26850*), which contains seven exons and is located on chromosome 4 of the thale cress (https://www.ncbi.nlm.nih.gov/gene/828792). *VTC2* was the last discovered gene of the L-galactose pathway for AsA biosynthesis. To identify the *VTC2* gene and other genes of the biosynthetic pathway, it was fundamental to have a collection of AsA-deficient *vtc* (for vitamin C) thale cress mutants. This collection of mutant plants was obtained from seeds of thale cress mutagenized with ethyl methanesulfonate (Conklin et al., 1996; Conklin et al., 2000). From the collection, mutant thale cress plants named *vtc2-1*, *vtc2-2*, and *vtc2-3* were characterized by their high sensitivity to the air pollutant ozone, which causes oxidative stress in plants. The high sensitivity to ozone is because the mutant plants had low levels of AsA in mature leaves, siliques, and inflorescences (Conklin et al., 2000). As part of these preliminary studies, the researchers determined that the *vtc2* mutants are conferred by a single monogenic recessive trait, and their locus is located on chromosome 4 (≈3 cM of the centromere) (Conklin et al., 2000). These results suggested that the *VTC2* gene-encoded protein participates directly or indirectly in the biosynthesis of AsA. In an attempt to characterize the mutant *VTC2* gene, a research team used a map-based cloning approach and determined that the *VTC2* gene is the same that the gene *At4g26850* (Jander et al., 2002). The sequence of the cloned gene *At4g26850* showed that it encoded a novel protein. Consequently, at the moment of their discovery, the researchers were unable to demonstrate if the encoded protein functions as a regulatory protein or has a catalytic function in the metabolism of AsA (Jander et al., 2002).

Furthermore, to determine the function of the encoded protein by the *VTC2* gene from thale cress and its homologous gene from *Actinidia chinensis* “kiwifruit”, Bulley’s research team used several *in silico*, *in vitro*, and *in vivo* approaches (Laing et al., 2007). Based on their results, the researchers concluded that the thale cress and kiwifruit genes are orthologous and encode the missing enzyme of the L-galactose pathway of AsA biosynthesis. This enzyme is best described as a GDP-L-galactose-hexose-1-phosphate guanylyltransferase (GGP), transferring a guanylate moiety (GMP) from GDP-L-galactose to a hexose 1-phosphate (Laing et al., 2007). The GGP enzyme contains an HxHxQ motif that is characteristic of the D-galactose-1-phosphate uridylyltransferase (GalT) family of the histidine triad (HIT) superfamily (Brenner, 2002). This enzyme converts GDP-L-galactose into L-galactose 1-phosphate and has a fundamental role in the L-galactose pathway because it catalyzes the committed step for AsA biosynthesis. In other words, the GGP enzyme makes the first catalytic reaction that channels the metabolic intermediates exclusively for the biosynthesis of AsA (Laing et al., 2007).

The recombinant GGP enzyme encoded by the *VTC2* gene of thale cress show some biochemical characteristics. First, the enzyme is monomeric with ≈55 kDa of molecular weight. To make its catalytic activity, the GGP enzyme does not require magnesium ions and employs D-mannose 1-phosphate as a better guanyl acceptor than Pi or PPi. Additionally, the GGP enzyme can use various hexoses 1-phosphate with configurations D or L as guanyl acceptors, such as β-D-glucose 1-phosphate (0.24 nmol.s^-1^.μg^-1^ protein), D-mannose 1-phosphate (0.33 nmol.s^-1^.μg^-1^ protein), α-D-glucose 1-phosphate (0.35 nmol.s^-1^.μg^-1^ protein), D-galactose 1-phosphate (0.38 nmol.s^-1^.μg^-1^ protein) and L-myoinositol 1-phosphate (0.42 nmol.s^-1^.μg^-1^ protein). The estimated *K*_cat_ value for the recombinant enzyme is ≈20 s^−1^(Laing et al., 2007).

Another research team that participated in the competition to discover the function of the encoded protein by the *VTC2* gene of thale cress was Clarke’s research team (Linster et al., 2007). These researchers provided additional information and reported a slightly different result than the previously described recombinant GGP enzyme by Bulley’s research team (Laing et al., 2007). These authors show that the GGP enzyme of thale cress is well-conserved in both animals and plants and belongs to a HIT superfamily of the GalT/Apa1 branch. Also, it was shown that the GGP enzyme presents an HLHPQ motif. In this HIT motif, the second histidine residue (H238) is responsible for attacking the nucleoside monophosphate moiety of substrates (i.e., GDP-L-galactose) by the formation of a covalent nucleotidylated enzyme intermediate. Furthermore, the covalent enzyme intermediate suffers a phosphorolysis reaction with inorganic phosphate. This phosphorolysis process releases the GDP moiety and the GGP enzyme. Then, the free GGP enzyme binds to another substrate molecule to start a new catalytic cycle. In summary, the GGP enzyme converts GDP-L-galactose reversibly into L-galactose 1-phosphate in a reaction that requires inorganic phosphate with the concomitant releasing of GDP (Linster et al., 2007). The biochemical properties of this recombinant GGP enzyme are: the molecular weight of the recombinant His-tagged enzyme is 53.1 kDa; the *K*_M_ values for GDP-L-galactose, GDP-D-glucose, and GDP-D-mannose are 10.0, 4.4, and 520 μM, respectively; the *K*_cat_ values for GDP-L-galactose, GDP-D-glucose, and GDP-D-mannose are 64.0, 23.0, and 0.093 s^-1^, respectively; and the *K*_cat_/*K*_M_ values for GDP-L-galactose, GDP-D-glucose, and GDP-D-mannose are 6.3x10^3^, 5.7x10^3^ and 1.9x10^-1^ mM^-1^.s^-1^, respectively. GGP activity also is measured in the reverse direction of catalysis by incubating the enzyme without inorganic phosphate in high concentrations of GDP and hexose 1-phosphates and measuring the production of GDP-hexoses (Linster et al., 2007).

Moreover, Smirnoff’s research team (Dowdle et al., 2007) reported that thale cress possesses a second gene named *VTC5* (*At5g55120*), which is a homolog to the *VTC2* gene (66% identical) but shows low levels of gene expression (from 100- to 1000-fold lower than *VTC2*). The *VTC5* gene contains seven exons and is located on chromosome 5 (https://www.ncbi.nlm.nih.gov/gene/835603). This gene encodes a second GDP-L-galactose phosphorylase with similar biochemical characteristics to the *VTC2-*encoded enzyme (Dowdle et al., 2007). As the AsA levels of the *vtc2-1* and the *vtc5* (*vtc5-1* and *vtc5-2*) mutants have ≈20% and ≈90%, respectively, of the wild-type, the researchers furthermore verified the function of the *VTC2* and *VTC5* genes in AsA biosynthesis by constructing double mutants (*vtc2-1* x *vtc5-1* and *vtc2-1* x *vtc5-2*). The seeds of homozygous double mutants from each F_2_ progeny have normal germination, but the seedlings stop growing up after the initial expansion of the cotyledons, which then bleached within two weeks. These perishing seedlings are rescued when they are transferred to a medium supplemented with AsA or L-galactose. L-galactose is a metabolic intermediary that is generated downstream of the reaction catalyzed by the GGP enzyme in the L-galactose pathway. In summary, the results of these genetic experiments indicate that the GGP enzyme, and consequently the L-galactose pathway, is the unique physiologically relevant biosynthetic pathway of AsA in thale cress seedlings (Dowdle et al., 2007).

The recombinant GGP enzymes encoded by the *VTC2* and *VTC5* genes of thale cress that were expressed in *Escherichia coli* presented some biochemical characteristics. First, the molecular weights are 48.9 and 48.3 kDa, respectively. Second, the *K*_M_ values for GDP-L-galactose are 250 and 667 μM, respectively. Third, the *K*_M_ values for phosphate are 251 and 130 μM, respectively. Fourth, the *K*_cat_ values for GDP-L-galactose are 2.0 and 2.7 s^-1^, respectively. Fifth, the *K*_cat_/*K*_M_ values for GDP-L-galactose are 8.2x10^-6^ and 4.0x10^-6^ mM^-1^.s^-1^, respectively. Sixth, the *K*_cat_/*K*_M_ values for phosphate are 8.1x10^-6^ and 20.6x10^-6^ mM^-1^.s^-1^, respectively. Finally, both recombinant enzymes have a substrate specificity of 100% for GDP-L-galactose and lower substrate specificity (from 0.1 to 3.7%) for UDP-D-glucuronic acid, UDP-D-galactose, ADP-D-glucose, UDP-D-glucose, and GDP-D-mannose (Dowdle et al., 2007).

### 3.6 L-galactose 1-phosphate phosphatase (GPP: EC 3.1.3.25)

L-galactose 1-phosphate phosphatase is encoded by the *VTC4* gene (*At3g02870*), which contains twelve exons and is located on chromosome 3 of the thale cress (https://www.ncbi.nlm.nih.gov/gene/821206). The *VTC4* gene was identified in a thale cress mutant called *vtc4-1*, which was isolated from a collection of AsA-deficient thale cress mutants (Conklin et al., 2000). The *vtc4-1* thale cress mutant is a low AsA producer plant, showing in their tissues (i.e., mature leaves, green siliques, and inflorescence) until ≈50% lower content of AsA compared with the wild-type plants (Conklin et al., 2000). It was proved that the *vtc4-1* thale cress mutant is conferred by a single monogenic recessive trait named *VTC4* locus, which is located on chromosome 3 (Conklin et al., 2000). Furthermore, using genetic mapping and DNA sequencing approaches was demonstrated that the *VTC4* locus corresponds to the *At3g02870* gene, and it was predicted that the gene encodes the enzyme L-galactose 1-phosphate phosphatase (Conklin et al., 2006). Subsequently, it was proved that the mutant *VTC4* gene has a transition mutation (C → T) at nucleotide +275 relative to the start codon. This mutation changes the amino acid sequence of the encoded GPP enzyme (P92L) within a well-conserved β-bulge of myo-inositol monophosphatases. Consequently, it was suggested that the mutation disrupts the localization of key catalytic amino acid residues within the enzyme active site. These catalytic amino acid residues could be involved in the interaction with the enzyme cofactor (Mg^2+^) and the substrate L-galactose 1-phosphate. These structural changes of the mutant GPP enzyme significantly affect its catalytic activity, decreasing it by ≈50% compared with the wild-type enzyme (Conklin et al., 2006).

GPP is a homodimeric enzyme belonging to the FIG (FBPase/IMPase/GlpX-like domain) superfamily of metal-dependent phosphatases. The enzyme catalyzes the Mg^2+^-dependent hydrolysis of the substrate L-galactose 1-phosphate (L-gal-1-p) to produce L-galactose. The partially purified enzyme from the young berry of *Actinidia deliciosa* “kiwifruit” is a homodimer with ≈65 kDa of molecular weight and has optimal catalytic activity at pH 7.0. The *K*_M_ value for L-gal-1-p depends on magnesium chloride concentration, spanning from 22 μM (at 4.8 mM MgCl_2_) to 41 μM (at 1.8 mM MgCl_2_). This magnesium-dependent enzyme activity has a *K*_a_ (Mg^2+^) of 200 μM, but a high concentration of magnesium ions (>2,000 μM) inhibits the enzyme, and the apparent *K*_i_ (Mg^2+^) is 460 μM. The partially purified enzyme from shoots of *Arabidopsis thaliana* “thale cress” has similar kinetic parameters to the kiwifruit enzyme, but some differences exist. Accordingly, the optimal pH for catalytic activity fluctuates from 6.8 to 7.0 (at 2 mM MgCl_2_ and 0.5 mM of L-gal-1-p), the *K*_M_ value for L-gal-1-p is 44 μM (at 2.0 mM MgCl_2_), the *K*_a_ (Mg^2+^) is 16 μM, and the apparent *K*_i_ (Mg^2+^) is 620 μM (Laing et al., 2004a).

Moreover, Laing’s research team obtained interesting results when functionally characterized the recombinant enzyme from kiwifruit expressed in *Escherichia coli* (Laing et al., 2004a). Similar to the partially purified enzymes from kiwifruit and thale cress, the recombinant enzyme is a homodimer with ≈70 kDa of molecular weight. In contrast to the partially purified enzymes from kiwifruit and thale cress that only are specific for the substrate L-gal-1-p, the recombinant one possesses phosphatase activity with both substrates L-gal-1-p and D-myo-inositol 1-phosphate (D-myo-1-p) but is ≈14 times more active with L-gal-1-p than the second one. Similar to the partially purified enzymes, the optimal pH for catalytic activity is 7.0. Also, on its kinetic parameters, the recombinant enzyme has different properties than the partially purified ones; accordingly, the high *K*_M_ values for L-gal-1-p and D-myo-1-p are 150 μM and 330 μM, respectively. The *K*_a_ (Mg^2+^) is 470 μM, and the apparent *K*_i_ (Mg^2+^) is 13,400 μM. The highest *K*_i_ (Mg^2+^) value indicates that the recombinant enzyme is more refractory to inhibition by magnesium ions than the enzymes extracted from the plants. Additionally, Laing’s research team shows that the L-galactose-1-phosphate phosphatase activity of the recombinant enzyme is inhibited by high concentrations of LiCl (*K*_i_ (Li^+^) = 3,700 μM) (Laing et al., 2004a). However, the kinetic analysis aforementioned is incomplete due to the lack of *K*_cat_ and inhibition kinetics to make a better comparison between enzymes. Also, it is necessary to evaluate the type of enzymatic inhibition. Finally, it has not been assessed if AsA (the final product of the L-galactose pathway of AsA biosynthesis) is a feedback inhibitor for this enzyme.

Similar assays were conducted by Gillaspy’s research team, whom functionally characterized the recombinant enzyme from thale cress. Their results show that the GPP enzyme is a moderately promiscuous enzyme that hydrolyses C1-monophosphorylated six-membered ring substrates such as L-gal-1-p, D-myo-1-p, and D-myo-inositol 3-phosphate (D-myo-3-p). The enzyme also hydrolyses a gama of substrates such as glycerol 2-phosphate, α-D-glucose 1-phosphate, D-galactose 1-phosphate, D-mannitol 1-phosphate, adenosine 2’-monophosphate, α-D-glycerophosphate, D-fructose 1-phosphate, and D-sorbitol 6-phosphate with a rate of activity in the range from 1.7 to 52.0%. The optimal pH for the catalytic activity of this enzyme is 7.5. Also, the enzyme requires MgCl_2_ as a cofactor in such a way that a concentration from 3,000 to 4,000 μM is necessary to activate the enzyme until 3-fold higher compared with the activity without the enzyme cofactor. This recombinant enzyme is inhibited for high concentrations of MgCl_2_ (>5 mM). Regarding its catalytic properties, the enzyme has an apparent *K*_M_ value of 107 μM and 191 μM for L-gal-1-p and D-myo-3-p, respectively. High concentrations (> 600 μM) of the substrate D-myo-3-p have an inhibitory effect on the enzyme. Based on the capability of the recombinant enzyme to use L-gal-1-p and myo-inositols, the researchers conclude that GPP is a bifunctional enzyme that could participate in both the L-galactose pathway and the myo-inositol pathway to biosynthesize AsA (Torabinejad et al., 2009).

### 3.7 L-galactose dehydrogenase (GDH: EC 1.1.1.117)

L-galactose dehydrogenase is encoded by the *GDH* gene (*At4g33670*), which contains five exons and is located on chromosome 4 of the thale cress (https://www.ncbi.nlm.nih.gov/gene/829509). In tomato, the *GDH* gene is located on chromosome 1 (Zou et al., 2006). The genome of both plant species has a unique copy of the *GDH* gene (Gatzek et al., 2002; Zou et al., 2006). Furthermore, several studies have demonstrated that the encoded enzyme of the *GDH* gene is a key player in the AsA biosynthesis by plants. Thus, when the *GDH* gene is suppressed using an antisense approach, the AsA content in thale cress tissues exposed to high light irradiation decreases significantly (Gatzek et al., 2002), confirming so the role of the GDH enzyme for AsA biosynthesis in plants, as has been previously demonstrated the late 1990s by Smirnoff’s research team (Wheeler et al., 1998).

The GDH enzyme catalyzes the penultimate step of AsA biosynthesis, which is the oxidation of L-galactose at position C1, transforming it into L-galactono-1,4-lactone. Smirnoff’s research team discovered the novel and unique GDH enzyme in cell-free extracts from leaves of thale cress and *Pisum sativum* “pea” embryonic axes, which promotes the L-galactose-dependent reduction of NAD^+^ (Wheeler et al., 1998). The discovery of the GDH enzyme was fundamental to the proposal of the L-galactose pathway for AsA biosynthesis in plants by Smirnoff’s research team (Wheeler et al., 1998).

Structurally, GDH is a monomeric enzyme dominated by a (β/α)8-barrel fold that belongs to the aldehyde-keto reductase (AKR) protein superfamily. It has eight parallel β-strands alternated with eight α-helices that run antiparallel in relation to the strands, forming the classical barrel-type fold. In the protein, N-terminus contains a beta-hairpin (β1 and β2) that shapes the bottom of the barrel and has a well-conserved cofactor binding region for NAD^+^. The enzyme has the typical conserved catalytic tetrad of this protein superfamily, which are Asp57, Tyr62, Lys90, and His127 in *Spinacia oleracea*. These amino acid residues also are involved in the direct interaction with NAD^+^, suggesting that this enzyme uses the same catalytic mechanisms as AKR, favoring the dehydrogenation of its substrate and reduction of NAD^+^ to NADH + H^+^ (Vargas et al., 2022).

The GDH enzyme shows some biochemical characteristics depending on the plant source and if the enzyme is native or recombinant. In thale cress and pea the native GDH enzyme is soluble with no obvious transit sequences, suggesting that the enzyme is located in the cell cytoplasm (Gatzek et al., 2002). The recombinant GDH enzymes from some plant species are monomeric, with molecular weights in the range from 34.2 to 40.0 kDa (Gatzek et al., 2002; Mieda et al., 2004; Laing et al., 2004b; Momma and Fujimoto, 2013; Vargas et al., 2022). However, the native one from pea is a homotetramer with a molecular weight of 156 kDa (the subunit molecular weight is 40 kDa), whereas the recombinant one from thale cress has monomeric and homodimeric conformations with molecular weights of 42.4 and 87.5 kDa, respectively (Gatzek et al., 2002). The optimal catalytic activity of the recombinant enzymes is in the pH range from 7.0 to 9.3 (Gatzek et al., 2002; Mieda et al., 2004; Laing et al., 2004b; Vargas et al., 2022). Regarding to the enzyme kinetic parameters, the reported *K*_M_ value for L-galactose is variable from 85 to 300 μM (Wheeler et al., 1998; Gatzek et al., 2002; Mieda et al., 2004; Laing et al., 2004b; Vargas et al., 2022). As well, other kinetic parameters reported for the recombinant GDH enzyme from camu-camu and spinach show some differences; thus, the *K*_cat_ values are 4.3 and 1.2 s^-1^, respectively, and the *K*_cat_/*K*_M_ values are 20.7 and 9.1 mM^-1^.s^-1^, respectively (Vargas et al., 2022). Finally, Gatzek et al., hypothesized that the GDH enzyme has simple kinetic characteristics, suggest that this enzyme does not has a regulatory property in the L-galactose pathway (Gatzek et al., 2002).

### 3.8 L-galactono-1,4-lactone dehydrogenase (GLDH: EC 1.3.2.3)

L-galactono-1,4-lactone dehydrogenase is encoded by the *GLDH* gene (*At3g47930*), which contains six exons and is located on chromosome 3 of the thale cress (https://www.ncbi.nlm.nih.gov/gene/823948). In tomato, the *GLDH* gene is located on chromosome 10, and the genome of this plant species harbor a unique copy of the *GLDH* gene (Zou et al., 2006). Previous investigations showed that the expression levels of the *GLDH* gene influence the biosynthesis and accumulation of AsA in plants. In this regard, Esaka’s research team generated AsA-deficient transgenic tobacco BY-2 cell lines (named AS1–1 and AS2–2) expressing antisense RNA that strongly inhibits the expression of the sense *GLDH* mRNA. Both transgenic tobacco BY-2 cell lines had a reduced quantity of mRNA molecules of the *GLDH* gene and a significant decline (from 22.6 to 25.6% lower than the wild-type cells) in the catalytic activity of the GLDH enzyme. Also, the transgenic cell lines showed from 24% (in AS1-1) to 27% (in AS2-2) less AsA content than the wild-type tobacco BY-2 cells (Tabata et al., 2001). Additionally, it was proved that thale cress seedlings of the SALK_060087 line, which carries a T-DNA insertion in the *GLDH* gene, do not develop beyond the cotyledon stage without adding AsA (Pineau et al., 2008). Also, more recently, it was demonstrated that the thale cress homozygous mutant (T-DNA insertion mutant designated *GLDH-236OE*), which overexpress the *GLDH* gene, has a significantly high content of AsA in their leaves than wild-type plants. Also, these *GLDH* gene-overexpressing mutant plants have more tolerance to high light due to a better capacity to eliminate reactive-oxygen species, absorb extra light, and dissipate the thermal energy (Zheng et al., 2019). Together, these results corroborate that the catalytic activity of the GLDH enzyme is an essential step in the L-galactose pathway for AsA biosynthesis in plants.

The GLDH enzyme is a monomeric aldonolactone oxidoreductase that belongs to the vanillyl-alcohol oxidase (VAO) flavoprotein family (Fraaije et al., 1998). The GLDH enzyme possesses two domains. The first one is a conserved FAD-binding domain, which binds the FAD cofactor non-covalently (Leferink et al., 2008b), and the second one is the CAP domain, which defines the substrate specificity and is responsible for its catalytic activity (Mattevi et al., 1997; Leferink et al., 2008a). This flavoenzyme catalyzes the last step of AsA biosynthesis and uses L-galactono-1,4-lactone (GL) and L-gulono-1,4-lactone as substrates to produce AsA. To catalyze this oxidoreduction reaction, GLDH, as a typical dehydrogenase, employs cytochrome c (Cyt c) as an electron acceptor, forming a transient (millisecond lifetime) low-affinity protein-protein complex (Hervás et al., 2013), but cannot use molecular oxygen as an electron acceptor because the enzyme has an Ala113 acting as a gatekeeper, which prevents the molecular oxygen from accessing to the isoalloxazine nucleus (Leferink et al., 2009a). This enzyme also can use phenazine methosulfate and 1,4-benzoquinone as electron acceptors (Mapson and Breslow, 1958; Leferink et al., 2008b).

Since its discovery, isolation, and initial characterization in the 1950s and furthermore, in the 1990s, the GLDH enzyme has been located in the plant mitochondria (Mapson, 1953; Mapson and Breslow, 1958; Ôba et al., 1994; Mutsuda et al., 1995). In these pioneering investigations was hypothesized that the GLDH enzyme is associated with components of the electron transport chain because oxidation of GL into AsA is inhibited by cyanide, azide, and CO in the dark (Mapson, 1953; Mapson et al., 1954; Mapson and Breslow, 1958). Furthermore, subcellular fractionation assays demonstrate that the GLDH enzyme is located on the inner mitochondrial membrane and GL oxidation delivers electrons to the mitochondrial electron transport chain between complexes III and IV, thus corroborating that Cyt c is the natural electron acceptor for this enzyme (Bartoli et al., 2000). More recently, GLDH has been established as an assembly factor for the proton-pumping Complex I (NADH:ubiquinone oxidoreductase) in the plant mitochondria (Schertl et al., 2012; Schimmeyer et al., 2016; Soufari et al., 2020).

Initial biochemical characterizations have tested that thiol-modifying agents inactivate the GLDH enzyme. These agents include o-iodosobenzoate, Cu^2+^ ions, and p-chloromercuribenzoate, suggesting that the GLDH enzyme requires amino acid residues with sulfhydryl groups for substrate binding (Mapson and Breslow, 1958). More recently, a highly conserved cysteine residue (C340) was identified, which is the redox-sensitive thiol, in the CAP domain of the GLDH enzyme (Leferink et al., 2009c). Additionally, was demonstrated that the amino acid residues G386 and R388 have essential roles in the active site of the GLDH enzyme. The first one binds the GL substrate, whereas the second one stabilizes the anionic state of the reduced FAD cofactor (Leferink et al., 2009b).

Also, the biochemical characterization of the enzyme shows some particularities. For example, the purified native GLDH enzyme from the root of *Ipomoea batatas* “sweet potato” is monomeric with a molecular weight of ≈56 kDa (Oba et al., 1995), similarly, the recombinant GLDH enzyme of thale cress show a molecular weight of ≈55 kDa (Leferink et al., 2008b). The native GLDH enzyme from sweet potato has optimal catalytic activity in the pH value from 7.4 to 7.9 (Oba et al., 1995), whereas the recombinant GLDH enzyme of thale cress has a broad pH range for activity with Cyt c from 8 to 9.5, with maximum activity at pH 8.8 (Leferink et al., 2008b). Similarly, the purified native GLDH enzyme from the florets of *Brassica oleracea* “cauliflower” showed an optimal pH value from 7.8 to 7.9 at 17 °C using Cyt c as an electron acceptor, whereas using phenazine methosulfate as an electron acceptor, the optimal pH was from 7.4 to 7.7 at 37 °C (Mapson and Breslow, 1958). Regarding substrate specificity, the purified native GLDH enzyme from cauliflower only can use as substrate GL but is unable to use L-gulono-1,4-lactone (Mapson and Breslow, 1958). Thus the *K*_M_ value of the native GLDH enzyme from cauliflower for the substrate GL is dependent on the electron acceptor, fluctuating from 2 mM (with Cyt c at pH 7.8) to 4 mM (with phenazine methosulfate at pH 7.4) (Mapson and Breslow, 1958). In contrast, the recombinant GLDH enzyme of thale cress can use both substrates GL and L-gulono-1,4-lactone but with different enzyme kinetic parameters. The *K*_M_ values are 170 and 13,100 μM, respectively, the *K*_cat_ values are 134 and 4.0 s^-1^, respectively, and the *K*_cat_/*K*_M_ are 7.7x10^2^ and 3.1x10^-1^ mM^-1^.s^-1^, respectively (Leferink et al., 2008b).

To date, significant gaps exist in our knowledge of the enzymes of the L-galactose pathway for AsA biosynthesis. It would be interesting to perform comparative studies at the functional and structural levels of the wild-type and mutant versions of the enzyme to understand its mechanisms of catalysis and regulation better. At the functional level, we should compare the enzyme kinetic parameters (e.g., *K*_M_, *K*_cat_, *K*_cat_/*K*_M_, *V*_max_, etc.). At the structural level, we could do a protein structure alignment of the wild and mutant versions of the enzyme with and without ligands (enzyme cofactor and substrates) to know the exact interaction and how this mutation affects both the structure and activity of the enzyme.

Also, in part, the marked differences in AsA accumulation within and between plant species can be attributed to the existence of mutant versions of the enzymes involved in these metabolic pathways and protein factors (e.g., transcription factors, proteasome components, transport of cofactors, etc.) regulating the activity of these enzymes.

## 4 Genetic strategies for regulation of AsA biosynthesis

### 4.1 Transcriptional regulation

**Figure 4:**
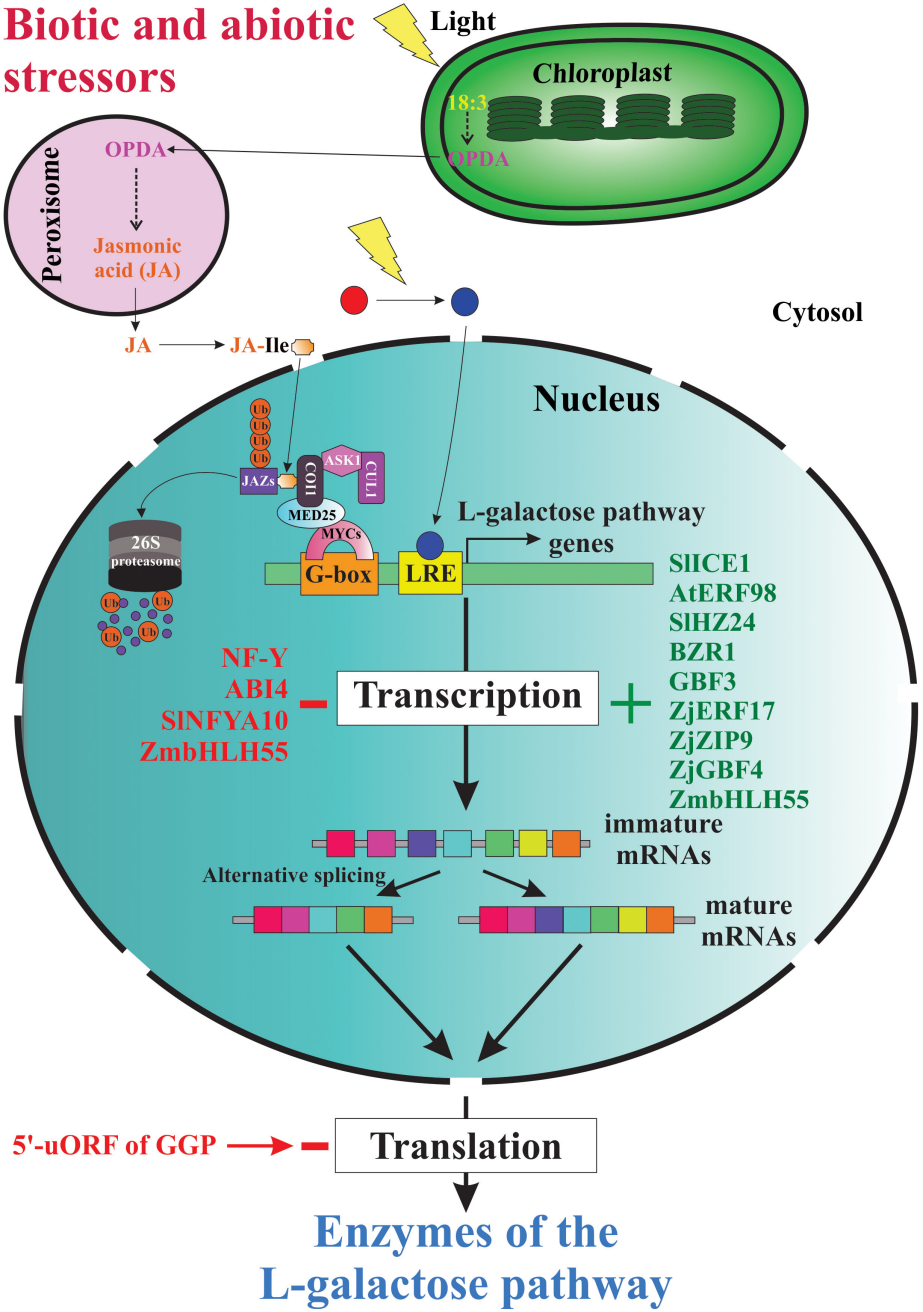
Genetic strategies for regulation of AsA biosynthesis in plants. When plants are under biotic and abiotic stress, they quickly up-regulate and/or down-regulate the expression of several genes involved directly or indirectly in AsA biosynthesis. Thus, several stressors induce the biosynthesis of jasmonic acid and other jasmonates, which starts intracellular signaling that finally induces the expression of AsA biosynthetic genes, translation of the corresponding enzymes, and increases the intracellular levels of AsA. The light, on its part, also starts an intracellular signaling activating transcription factors that bind with light-responsive promoter elements (LRE) and induces the transcription of gene-encoding enzymes of the L-galactose pathway. Until now, also were identified several transcription factors that activate (e.g., SlICE1, AtERF98, SlHZ24, BZR1, etc.) or inactivate (i.e., NF-Y, ABI4, SINFYA10, and ZmbHLH55) the transcription of the genes. In addition, alternative splicing is a probable mechanism involved in the regulation of AsA biosynthesis. Finally, the GGP gene is controlled *via* post-transcriptional repression by a conserved cis-acting upstream open reading frame (5’-uORF) present in the 5’-untranslated region of its mRNAs.

Since the elucidation of the main metabolic pathway for AsA biosynthesis in plants (Wheeler et al., 1998), the scientific curiosity to understand the genetic strategies used by plants to regulate the biosynthesis and accumulation of this vitamin has been strongly stimulated. According to published reports worldwide, the AsA content shows a great variability between plant species (Gallie, 2013; Williams et al., 2016; Castro et al., 2018; Aniceto et al., 2021), between cultivars and genotypes (Nishiyama et al., 2004; Rassam and Laing, 2005; Carneiro Ferreira et al., 2021), between tissues types (Rassam and Laing, 2005; Li et al., 2010; Castro et al., 2015a; Vidović et al., 2016), and between different developmental and fruit ripening stages (Pateraki et al., 2004; Ioannidi et al., 2009; Mellidou et al., 2012). To date, we know that the AsA content in plant tissues is a result of a dynamic equilibrium controlled by a complex and little-comprehended mechanism of regulation that orchestrates responses to biotic and abiotic environmental cues (e.g., high light intensity, oxidative stress, etc.) by enabling or disabling its anabolism, catabolism, recycling, and its intra- and intercellular transport and distribution (Bulley and Laing, 2016; Mellidou and Kanellis, 2017). Until now, our knowledge of the genetic strategies for regulating AsA biosynthesis used by plants at transcriptional and post-transcriptional levels is scarce, dispersed, and fragmentary (**Figure 4**).

Several studies show that the differential expression of genes encoding enzymes of the L-galactose pathways affects the AsA contents in tissues/organs of various plant species. The first study with *Malpighia glabra* “acerola” shows that the AsA content in ripening fruits decreases in its transition from unripe (150 μmol.g^-1^ fresh weight [FW]) to ripe (50 μmol.g^-1^ FW) fruits. This change in the AsA content of ripening fruits correlates with the abundance of mRNA transcripts from the genes *MgGMP*, *MgGME*, *MgGGP*, *MgGDH*, and *MgGLDH* (Badejo et al., 2009b). Similarly, the AsA content in seeds (from ≈0.18 to ≈0.03 μmol.g^-1^ FW) during the ripening process correlated with the mRNA levels of the five genes evaluated. Also, this research shows that the expression levels of the five genes in fruits are highest compared to seeds (Badejo et al., 2009b). Additionally, a comparison of the AsA contents and abundance of mRNA molecules of the L-galactose pathway in leaves of acerola (AsA content =18.86 ± 0.95 μmol.g^-1^ FW) and thale cress (AsA content = 2.50± 0.40 μmol.g^-1^ FW) shows that the five genes evaluated have highest mRNA transcripts levels in acerola (from 5 to 700-fold higher expression levels) compared to thale cress (Badejo et al., 2009b). A second study with *Brassica campestris* “non-heading Chinese cabbage” shows that the cultivar Suzhouqing has significantly higher total AsA content in leaves (≈1.1 mg.g^-1^ FW) in comparison to petioles (≈0.4 mg.g^-1^ FW), and roots (≈0.2 mg.g^-1^ FW). When was measured the expression levels of genes of the L-galactose pathway in the three tissues, the researchers recorded the highest expression levels of the genes *BcPMI1-2, BcPMM1-2, BcGMP1-3, BcGME1-2, BcGGP1-4, BcGPP, BcGDH, and BcGLDH* in leaves in comparison to petioles and roots (Ren et al., 2013). A third study compared the expression levels of genes of the L-galactose pathway in *Citrus sinensis* “navel orange” and *Citrus unshiu* “satsuma mandarin”, which have contrasting AsA concentrations in fruit peels and fruit pulps. Thus, fruit peels (flavedo) of ripe fruits of oranges and mandarins contain 2.40 and 1.50 mg.g^-1^ FW of AsA, respectively. However, pulps of ripe fruits of oranges and mandarins contain 0.50 and 0.20 mg.g^-1^ FW of AsA, respectively. The higher AsA content in fruit peels of oranges compared to mandarins is related to the significant increase of mRNA transcripts of the genes *GMP*, *GGP*, *GPP*, and *GLDH* in at least one of the last three months of the fruit ripening process. Similarly, the major AsA content in fruit pulps of oranges compared to mandarins is related to the significant increase in expression levels of the genes *GGP* and *GPP* during the last six months of the fruit ripening process (Alós et al., 2014). Together, the results of this research show that the mRNA relative abundance of the six genes (*GMP*, *GME*, *GGP*, *GPP*, *GDH*, and *GLDH*) was higher in fruit peels (from 0.5 to 25-fold) compared to fruit pulps (from 0.1 to 3-fold), suggesting that the AsA content in both fruit structures depends of the expression levels of the genes encoding enzymes of the L-galactose pathway (Alós et al., 2014). Finally, it was demonstrated that differences in the AsA content in leaves and unripe fruits (fruit pulp and fruit peel) of two genotypes of camu-camu (*Md*-60,06 and *Md*-02,04) correlate with the differential expression of genes encoding enzymes of the L-galactose pathway. The fruit peel (*Md*-60,06 = 165 μmol.g^-1^ FW; *Md*-02,04 = 95 μmol.g^-1^ FW) contains ≈ 1.5-fold more AsA than the fruit pulp (*Md*-60,06 = 125 μmol.g^-1^ FW; *Md*-02,04 = 55 μmol.g^-1^ FW), and ≈15-fold more AsA than the leaves (*Md*-60,06 = 12 μmol.g^-1^ FW; *Md*-02,04 = 5 μmol.g^-1^ FW). The mRNA relative abundance of the genes *MdGMP*, *MdGME*, *MdGGP*, *MdGPP*, *MdGDH*, and *MdGLDH* is higher in fruit peels and fruit pulps (from 2- to 5-fold more) compared to leaves (Castro et al., 2015a). Additionally, other investigations corroborate that the differential expression of genes encoding enzymes of the L-galactose pathways influences the AsA contents in tissues of other plant species such as *Actinidia eriantha* “kiwifruit” (Jiang Z.-Y. et al., 2018), *Apium graveolens* “celery” (Huang et al., 2016), Brassica rapa “rape mustard” (Duan et al., 2016), *Camellia sinensis* “tea” (Li et al., 2017), *Malpighia glabra* “acerola” (Suekawa et al., 2019), *Physcomitrium patens* “moss” (Sodeyama et al., 2021), *Prunus avium* “sweet cherry” (Liang et al., 2017), and *Vaccinium corymbosum* “blueberry” (Liu F. et al., 2015). Together, the results of the investigations mentioned show a common pattern, which is the direct correlation between the expression level of the genes encoding enzymes of the L-galactose pathway and the AsA content in the tissues/organs of several plant species. Thus, when the expression of these genes increases, the AsA content increases in the corresponding plant tissue/organ, and viceversa.

An environmental key player that influences the biosynthesis and accumulation of AsA in plants is light. Because the biosynthesis of AsA is a light and photosynthesis-dependent metabolic process. Since the light boosts the photosynthetic process, which provides the sugar precursors (i.e., hexoses) required for the *de novo* biosynthesis of AsA. Light also plays an alternative function as a signal for regulating AsA biosynthesis in the leaves of thale cress (Yabuta et al., 2007). According to this, when 2-week-old thale cress plants grown under a 16 h daily photoperiod (total AsA content in leaves is ≈5 μmol.g^-1^ FW) are moved into darkness condition for 72 h, the AsA content in its leaves decreases by 91% (total AsA content is ≈0.45 μmol.g^-1^ FW), whereas plants exposed to continuous illumination, in the same period, show an increase in the AsA content in its leaves by 171% (total AsA content is ≈8.55 μmol.g^-1^ FW). The mRNA levels of the genes *AtGME* and *AtGDH* show similar patterns of variation under light and darkness conditions. However, the mRNA levels of the genes *AtGMP*, *AtGPP*, *AtGGP*, and *AtGLDH* are down-regulated under darkness and up-regulated under a continuous illumination (Yabuta et al., 2007). Also, it was demonstrated that when acerola plants are grown in darkness, the AsA content in leaves decreases significantly from 50% to 65% after four and seven days, respectively. This decrease in AsA content was related to the down-regulation in the expression of the genes *MgGME*, *MgGGP*, *MgGDH*, and *MgGLDH*, with the exception of the gene *MgGMP*. However, when the plants are exposed to illumination for three days, the AsA content in leaves increased at levels similar to plants growing under a normal regime of light and dark conditions. This increase in AsA content in leaves is related to the up-regulation in the expression of the genes encoding enzymes of the L-galactose pathway, but the expression levels of the *MgGMP* gene is reduced until approximately the levels that it had before the treatment under darkness (Badejo et al., 2009b). Another study with *Solanum lycopersicum* “tomato” showed that plants totally shaded (only received 29% of incident radiation) during seven days have a reduced content of AsA in their leaves (2.1 g.kg^-1^ DW). In contrast, sun-exposed plants in the same period have a major content of AsA in their leaves (5.8 g.kg^-1^ DW). Thus, plants grown under shading have a significant reduction (≈66%) in their AsA content in leaves. This reduction in the AsA content of leaves under shading was correlated with reduced mRNA levels of the genes *SlGMP1*, *SlGMP3*, *SlGME1*, *SlGME2*, *SlGGP*, *SlGPP1*, and *SlGPP2*. The *SlGME1* gene has the more noticeable down-regulation because the quantity of its mRNA transcripts is reduced 13-fold after seven days under shading conditions (Massot et al., 2012). Also, in *Brassica rapa “*rape mustard” (NHCC cultivar), when are under 24 h in darkness, the AsA content in leaves decreased (≈90%), but when plants are exposed to continuous illumination for 24 h, the AsA content increases by 180%. These fluctuations in the AsA content are related to the differential expression of the genes encoding enzymes of the L-galactose pathway. Thus, under darkness conditions, all the genes (*BracPMI1, BracPMMa-c, BracGMPa-c, BracGMEa-b, BracGGPa-c, BracVTC5, BracGPP, BracGDH, and BracGLDH*) are down-regulated, but under illumination, all these genes are up-regulated (Duan et al., 2016). Also, a recent study shows that when one-week-old protonemata of *Physcomitrium patens* “spreading earthmoss” is moved to darkness conditions for 2 days and then grown under continuous illumination (100 μmol photons.m^−2^.s^−1^), the total AsA content increases 3-fold within 12 h (from 0.92 to 2.80 μmol.g^-1^ FW). The increase in AsA content is related to induction, in the first three hours, in the transcription of the genes *PpPMI1-2*, *PpPMM*, *PpGMP1-2*, *PpGME1*, *PpGGP1-2*, *PpGPP1-2*, *PpGDH1*, and *PpGLDH*. However, some ortholog genes show no or low levels of gene expression under illumination. For instance, among the *PpGME* ortholog genes, *PpGME2* and *PpGME3* are unresponsive to light. The same expression behaviour shows the *PpGGP3* ortholog gene (Sodeyama et al., 2021). In summary, these results suggest that light controls the biosynthesis and accumulation of AsA in leaves of various plant species by inducing the transcription of the genes encoding enzymes of the L-galactose pathway.

The ability of the genes encoding enzymes of the L-galactose pathway to respond to a light stimulus is because these genes contain light-responsive cis-acting elements in their promoters. According to this, a bioinformatic analysis of genes from thale cress identified 16 light-responsive promoter motifs (e.g., ACA, ACE, ATCT, BoxII, BoxIII, G-box, GATA, LAMP, CTC, etc.). All genes of the L-galactose pathway (except the *AtGLDH* gene) contain one (*AtPMM* gene), two (*AtGPP* gene), three (*AtGDH* gene), four (*AtPMI, AtGMP*, and *AtGME* genes) or six (*AtGGP* gene) light-responsive promoter motifs (Ioannidi et al., 2009). Similar light-responsive promoter motifs contain the genes involved in the AsA biosynthesis of *Solanum melongena* “eggplant”. The genes *SmGMP*, *SgME1-2*, *SgGGP*, *SmGPP*, *SmGDH*, and *SmGLDH* significantly increase their expression levels when exposed to light. The light response capability of these genes is related to cis-acting elements contained in their promoters. Among these light-responsive promoter motifs identified in all genes appear ARE, AE-box, AT1-motif, CATT-motif, GA-motif, GAG-motif, I-box, TCCC-motif, and others (Jiang M. et al., 2018). Finally, it was demonstrated that when *Cucumis sativus* “cucumber” plants are exposed to UV-B radiation, the total AsA content in leaves increases significantly. Thus, the content of total AsA increased by 1.41-fold at 20 hours of UV-B exposure. This significant increase in AsA content in leaves is related to increased mRNA transcripts of the *CsGLDH* gene. The induction in the expression of this gene starts during the first hour of UV-B exposure and is induced during the entire period of exposure. The highest mRNA transcript level (63-fold) is recorded at 16 hours of UV-B exposure. The ability of the *CsGLDH* gene to respond to the UV-B stimulus is attributed to the presence of cis-acting elements located in their promoter. These cis-acting elements identified in the gene belong to the light-responsive promoter motifs type. Among the cis-acting elements identified in the promoter of the *CsGLDH* gene are four I-Box (core sequence: GATAA), two CCAAT-Box (core sequence: CCAAT), and one ATCTA-motif (core sequence: ATCTA) (Liu et al., 2019). However, to corroborate the functionality of these light-responsive promoter motifs identified to date, it will be fundamental to validate them experimentally.

Until now, some light-responsive promoter motifs have been validated functionally. In this regard, it was demonstrated that in 2-week-old *Oryza sativa* “rice” the mRNA transcripts of the genes *OsGPP* and *OsGLDH* increase significantly in the rice shoots when the plants are grown under illumination, but both genes are down-regulated under darkness conditions. The ability of both genes to respond to light is due to the presence of light-responsive promoter motifs. The *OsGPP* gene contains in its promoter (region from -522 to -517) a well-conserved GT1 motif (GAAAAA). However, the *OsGLDH* gene contains in its promoter two well-conserved motifs. The first one is a GT1 motif (GGTAAA) located in the promoter from -374 to -369 bp region, whereas the second one is a TGACG motif located in the promoter from -522 to -517 bp region. To demonstrate that these light-responsive promoter motifs are involved in responses to light stimulus, the researchers used reporter constructs conformed by various lengths of the promoter region of the genes *OsGPP* and *OsGLDH* to control the expression of the firefly luciferase (*Fluc*) reporter gene (Fukunaga et al., 2010).

Similar methodological approaches, using the *Fluc* or *GUS* (β-glucuronidase) reporter gene systems, were applied to validate the function of light-responsive promoter motifs of other light-inducible genes encoding enzymes of the L-galactose pathway. For instance, the *AtGGP* gene (*VTC2*) of thale cress contains a critical cis-element for light regulation located in the promoter between the -70 and -40 bp region. This light-responsive promoter motif is novel because it does not have homologous sequences for known consensus elements for trans-acting factors (Gao et al., 2011). Also, the *AdGGP* and *AeGGP* genes of *Actinidia deliciosa* and *Actinidia eriantha*, respectively, also are light-inducible genes. In both genes were identified light-responsive promoter motifs such as ATC, Box-I, chs-CMA2b, and G-box (Li et al., 2013b). Additionally, it was demonstrated that the *AdGPP* gene of *Actinidia deliciosa* contains several light-responsive promoter motifs, including Box I, L-box, MBS, MRE, and a TCT motif (Li et al., 2013a). Again, as mentioned previously, it is necessary to conduct further analysis of these promoters and their interacting transcription factors to reveal the molecular mechanisms involved in regulating AsA biosynthesis by light in several plant species. Also, it is necessary to identify the components that constitute the molecular machinery involved in light reception, the intracellular signalosome, the activated/inactivated transcription factors that control the differential expression of both classes of genes involved in regulation or that encode enzymes of the L-galactose pathway.

In addition to light, when plants are under biotic or abiotic stress, a hormone-mediated response is activated to stimulate the *de novo* biosynthesis of AsA. Well-studied plant hormones involved in the complex stress response are jasmonates (e.g., jasmonic acid (JA), methyl jasmonate (MeJA), and other related compounds), which are lipid-derived hormones ubiquitous in plant intracellular signaling (Sasaki-Sekimoto et al., 2005). Thus, when plants are stressed with elicitors, wounding, osmolytes or ozone exposure, the intracellular levels of jasmonates increase due to their *de novo* biosynthesis. To understand the mechanisms of action of these hormones, several research teams have been using as a strategy the exogenous application of jasmonates to whole plants or specific organs. Thus, more than twenty years ago researchers demonstrated that exogenous application of MeJA induces AsA biosynthesis in plants (Maksymiec and Krupa, 2002). In this research when leaves of thale cress are treated with 100 μM of MeJA their AsA content increased more than 2-fold (from 1.65 ± 0.12 to 3.89 ± 0.21 μmol.g^-1^ FW). In addition to the AsA increase, the authors recorded a high hydrogen peroxide production (Maksymiec and Krupa, 2002). Similarly, when cellular suspensions of thale cress or tobacco Bright Yellow-2 (BY-2) are treated with MeJA, the *de novo* biosynthesis of AsA is stimulated. In BY-2 cells, the stimulation of AsA biosynthesis coincides with the transcriptional induction of at least two late MeJA-responsive genes including the *GME* gene and a putative L-gulono-1,4-lactone dehydrogenase/oxidase gene (Wolucka et al., 2005). Also, when 10-day-old thale cress seedlings are treated with 30 μM of jasmonic acid (JA), the up-regulation of jasmonate-responsive genes (JRGs) is achieved. Consistent with the cDNA microarray data, some JRGs belongs to the L-galactose pathway, including *VTC1* (*At2g39770* gene), *VTC2* (*At4g26850* gene), and *VTC5* (*At5g55120* gene) (Sasaki-Sekimoto et al., 2005). Also, when seedlings of *Agropyron cristatum* are exposed to 1 μM of JA for 24 h at 25 °C with a continuous light intensity of 200 μmol.m^-2^.s^-1^, the AsA content in leaves increases from 4.22 ± 0.24 to 6.59 ± 0.37 μmol.g^-1^ FW (Shan et al., 2011). The AsA increase is related to a ≈2-fold increase in transcript levels of the *GLDH* gene and a significant increase in the catalytic activity of the GLDH enzyme (water-treated seedlings = 2.00 ± 0.16 units.g^-1^ FW vs JA-treated seedlings = 3.90 ± 0.27 units.g^-1^ FW). In this research, it was also demonstrated that JA treatments increase the phosphorylation levels of mitogen-activated protein kinase kinase (MEK1/2), suggesting that the MAP kinase signaling pathway is involved in the modulation of the antioxidant defense by JA (Shan et al., 2011). In addition, it was demonstrated that since the first hour of treatment of thale cress with a 50 μM MeJA solution, the rosette leaves increases their AsA content on average 0.2 μmol.g^-1^ FW (≈7% increase in AsA content) compared with control plants (Suza et al., 2010). The AsA increase in MeJA-treatment plants is related to the up-regulation of several gene-encoding enzymes of the L-galactose pathway after six hours of treatment according to the Genevestigator gene expression data. These genes include *PMI*, *PMM*, *VTC1*, *GME*, *VTC2*, *VTC5*, and *GDH*. However, the *GLDH* gene is downregulated under MeJA-treatment (Suza et al., 2010).

Furthermore, several investigations were conducted to discover the players involved in the intracellular signaling to achieve the up-regulation of jasmonate-responsive genes. For example, the treatment of seedlings of *Agropyron cristatum* with JA increase the phosphorylation levels of mitogen-activated protein kinase kinase (MEK1/2), suggesting that the MAP kinase signaling pathway is involved in the modulation of the antioxidant defense by JA (Shan et al., 2011). Also, it was investigated the relationship between hydrogen sulfide (H_2_S) and MEK1/2 activation under JA-treatment to regulate the redox state of AsA in leaves of thale cress. The results indicate that JA significantly enhances the phosphorylation level of MEK1/2, induces the production of endogenous H_2_S and increases the ratio of reduced AsA to dehydroascorbate in wild type plants. The increase in AsA biosynthesis is related to up-regulation of the *GLDH* gene and to increased catalytic activity of their encoded enzyme (Shan et al., 2019). Similarly, it was investigated the relationship between MEK1/2 and nitric oxide (NO) in JA-regulated metabolism of AsA and glutathione in maize leaves. The results showed that JA induced the production of NO, enhanced the phosphorylation level of MEK1/2, increased the catalytic activity of the GLDH enzyme and enzymes of the ascorbate-glutathione pathway, then increasing the AsA and glutathione (GSH) contents. Together, these results suggest that in maize leaves, JA induces the phosphorylation and activation of MEK1/2 by the action of NO. Subsequently, the active MEK1/2 up-regulates AsA and glutathione metabolism in maize leaves (Shan and Sun, 2018). In addition, it was demonstrated that the intracellular signaling cascade started by jasmonates has as central components the E3 ubiquitin ligase SCF^COI1^ and Jasmonate ZIM-domain (JAZ) proteins (Katsir et al., 2008a; Pauwels and Goossens, 2011). These components repress the transcription of jasmonate-responsive genes. To activate this system an amino acid-conjugated form of JA, jasmonoyl-isoleucine (JA-Ile), binds and activates the F-box protein coronatine-insensitive 1 (COI1). Subsequently, activate COI1 induces the ubiquitin-dependent degradation of jasmonate ZIM domain (JAZ) proteins that repress transcription of jasmonate-responsive genes (Katsir et al., 2008b; Mach, 2009; Yan et al., 2009). Together, these results suggest that until have been identified some players of the complex intracellular signaling pathway started by jasmonates to respond to stressful conditions, so we have many knowledge-gaps of these key process.

Actually, some transcription factors (TFs) that regulate the expression levels of the genes encoding enzymes of the L-galactose pathways have been identified and functionally characterized. The TFs recognize specific cis‐acting elements and bind to promoters of target genes to control their expression. These TFs could be categorized as activators or inactivators of transcription. The first ones increase the transcript levels of their target genes, and the second ones decrease the transcript levels of their target genes. The first category includes the gene *ICE1* de *Solanum lycopersicum* “tomato” (*SlICE1*). This gene encodes a transcription factor that belongs to the basic helix-loop-helix (bHLH) DNA-binding superfamily protein. Transcripts of *SlICE1* are detected in several tissues of tomato (i.e., younger leaves, flowers, immature and mature fruit), and when it is overexpressed, the AsA accumulation increases, and the plants improve their chilling tolerance, but the activated target genes and their interacting target sequences for SlICE1, which could be involved in AsA metabolism, are undiscovered, and then the molecular mechanisms of action of this TF are unknown (Miura t al., 2012a; Miura et al., 2012b). Also, a gene encoding a type of ethylene response factor (ERF), which is characterized by its conserved DNA-binding domain and pertains to the AP2/ERF transcription factor superfamily (Nakano et al., 2006), was identified in *Arabidopsis thaliana* (*AtERF98*). Plants overexpressing the *AtERF98* gene have increased AsA levels, while plants having the mutant versions of the *AtERF98* gene (knockout and knockdown mutants) display reduced AsA contents. The researchers demonstrated that the regulation of the AsA content by AtERF98 is through the transcriptional activation of AsA biosynthesis-related genes. This transcriptional activation was proved using quantitative real-time PCR of *AtERF98*-overexpressing plant lines. These plant lines presented increased transcript levels of the genes *VTC1*, *VTC2*, *GDH*, and *GLDH*. Further, using transient expression and chromatin immunoprecipitation assays, the researchers tested that AtERF98 binds to a DRE-2 cis-element (ACCGAC) on the *VTC1* gene promoter (Zhang et al., 2012).

Additionally, Chinese researchers using a yeast one-hybrid assay and electrophoretic mobility shift assay (EMSA) identified an HD-Zip I family transcription factor of *Solanum lycopersicum*. This TF is named SlHZ24 and binds to a regulatory cis-element in the promoters of three genes of the L-galactose pathway. These genes encode GDP-D-mannose pyrophosphorylase 3 and 4 (SlGMP3 and SlGMP4), GDP-D-mannose 3’,5’-epimerase 2 (SlGME2), and GDP-L-galactose phosphorylase (SlGGP). The binding of SlHZ24 on the promoter of the AsA biosynthetic genes increases the AsA levels, suggesting that this TF positively regulates the accumulation of AsA. An additional and interesting discovery shown in this research is the fact that *SlHZ24* expression was light-dependent because their transcript levels quickly decrease under darkness and increase under illumination conditions (Hu et al., 2016). Another gene that boosts AsA accumulation in tomatoes is *BES1*, which encodes the brassinosteroid (BR) response transcription factor Brassinazole resistant 1 (BZR1) (Liu et al., 2014). This TF is a master regulator of the expression of multiple genes and thus has pleiotropic functions. When biotic and abiotic cues affect the plants, the precursor campesterol is converted to the phytohormone brassinosteroid, which subsequently activates BZR1. Activated BZR1 directly regulates the expression of thousands of downstream responsive genes (Lv and Li, 2020). Furthermore, two genes encoding activators TFs were identified in the fruit of *Actinidia eriantha*. The first gene encodes a 1R‐subtype myeloblastosis (MYB) protein, which interacts with the promoter of *GGP3* and activates its transcription. The overexpression and genetic edition of *MYBS1* increase the AsA levels. The second gene encodes the bZIP transcription factor GBF3 (a G‐box binding factor). This transcription factor forms a protein complex with MYBS1 bound to the promoter of *GGP3*. The interaction among MYBS1-GBF3-*GGP3* promoter additively promotes the transcription of the gene *GGP3* and boosts the AsA content. Moreover, the researchers have shown that the phytohormone abscisic acid represses MYBS1 but not GBF3, reducing both the *GGP3* expression and AsA levels (Liu et al., 2022).

Also, recently Chinese researchers using transcriptomic and metabolomic approaches during the fruit development of *Ziziphus jujuba*, identified some key candidate genes encoding TFs that could control the AsA level in the fruit of this plant species. The expression levels of these genes encoding TFs (*ZjERF17*, *ZjbZIP9*, and *ZjGBF4*) show high correlation coefficients (0.99, –0.92, –0.89, respectively) with AsA levels. Also, high correlation coefficient values show the expression levels of these TFs and some AsA biosynthesis-related genes. Together these results suggest that these three genes encoding TFs probably have key roles in regulating AsA biosynthesis (Lu et al., 2022).

Transcription factors that act as repressors of the expression of genes encoding enzymes of the biosynthesis pathway have been identified. For example, the gene *LEAFY-COTYLEDON1-LIKE4* (*L1L4*, *NF-YB6*) encodes a heterotrimeric nuclear transcription factor Y (NF-Y), which is a master regulator of some biosynthetic pathways. A mutant line (line 4) of *Solanum lycopersicum* bearing a disrupted version of this TF shows high AsA content in its fruit, suggesting that normally this gene downregulates the production of AsA. Again, in this study, as in the previous one, the inactivated target genes and their interacting target sequences for L1L4 are unknown, and thus the molecular mechanisms of action of this TF are unsolved (Gago et al., 2017). More recently, it was identified that ABA INSENSITIVE 4 (ABI4), which is a key factor in the abscisic acid (ABA) signalling pathway, directly binds to the −206 CCAC motif of the *VTC2* promoter, repressing the transcription of this gene and decreasing the AsA levels (Yu et al., 2019; Kakan et al., 2021). Also, an investigation that used binding assays in yeast and functional analyses in *Solanum lycopersicum* found a CCAAT-box transcription factor (SlNFYA10). This TF interacts with promoters of the *SlGME1* and *SlGGP1* genes and has a negative effect on the transcription of both genes. Thus, transgenic plants overexpressing *SlNFYA10* display the lowest levels of *SlGME1* and *SlGGP1* transcripts and decreased accumulation of AsA in their leaves and fruit (Chen et al., 2020).

In addition to the described TFs, which function as positive or negative regulators of transcription, recently, a TF of *Zea mays* was found that has both effects. This TF is a basic helix-loop-helix 55 (ZmbHLH55) that controls AsA biosynthesis in maize. The assays display that the protein ZmbHLH55 has a nuclear localization. Also, the TF forms homodimers that bind to DNA and has transactivation activity in yeast. Analysis of gene expression shows that ZmbHLH55 activates the expression of *ZmPGI2*, *ZmGME1*, *and ZmGLDH*; but negatively regulates the expression levels of the *ZmGMP1* and *ZmGGP* genes (Yu et al., 2021).

### 4.2 Post-transcriptional regulation

Alternative splicing is a well-known post-transcriptional regulatory mechanism of genes involved in plant metabolic pathways (Lam et al., 2022). It could be a common mechanism used by plants to regulate the intracellular levels of AsA. Thus, a recent transcriptomic analysis of *Actinidia chinensis* “kiwifruit” fruit at different days after pollination (i.e., 20, 120, and 127 days) found several alternative splicing events in genes encoding enzymes of the L-galactose pathway for AsA biosynthesis (Tang et al., 2016). The genes that show alternative splicing events are *GME* and *GPP*. Also, alternative splicing events were detected in other genes involved in the metabolism of AsA. These other genes include those encoding the enzymes glucose-6-phosphate isomerase, pectin methylesterase, mannose-6-phosphate isomerase, phosphomannomutase, L-ascorbate peroxidase, D-galacturonic acid reductase, inositol-3-phosphate synthase, polygalacturonase, and myo-inositol oxygenase. Finally, alternative splicing events were found in genes encoding enzymes of the AsA recycling pathway (ascorbate-glutathione cycle), including monodehydroascorbate reductase and dehydroascorbate reductase. This pathway is responsible for AsA regeneration from its oxidized forms, such as monodehydroascorbate and dehydroascorbate (Foyer and Noctor, 2011). Although alternative splicing events have been detected in some genes involved in the AsA metabolism of kiwifruit, to date, similar processes have not been reported in other plant species. Also, it is unknown whether these processes regulate AsA levels in the plant cells and whether they are present; the molecular mechanisms involved are unknown.

Another novel genetic strategy that controls AsA in plants was discovered some years ago by the Laing’s research team. These researchers prove that the AsA content is controlled *via* the post-transcriptional repression of the *GGP* gene by a conserved cis-acting upstream open reading frame (5’-uORF) present in the 5’-untranslated region of its mRNAs. This 5’-uORF encodes a peptide from 60 to 65 amino acid residues. Under high AsA content, this uORF suppresses the translation of the downstream main ORF (mORF) that encodes the GGP enzyme by causing a ribosomal stalling. When the researchers used point mutations or deletions of the 5’-uORF, the feedback regulation of translation by AsA did not work, increasing the AsA content in leaves. The authors suggest that the encoded peptide by the noncanonical 5’-uORF functions in the AsA inhibition of the translation (Laing et al., 2015), but still, the molecular mechanisms of this inhibitory effect are unresolved. According to some research in plants, the ribosomal stalling induced by 5’-uORF is a sequence-dependent process, repressing the synthesis of proteins encoded by the mORFs during both steps of ribosomal translation: elongation and termination (Hayashi et al., 2017). Another molecular mechanism proposed to explain the ribosome stalling phenomena is that these 5’-uORFs inhibit the mORF translation *via* the suggested “roadblock” mechanism, in which a stalled ribosome with a 5’-uORF blocks the ribosomal scanning, whereby avoiding the arrival of other ribosomes in the AUG codon of the mORF (Wang and Sachs, 1997).

## 5 Biochemical strategies for regulation of AsA biosynthesis1

### 5.1 Control of the enzyme levels

**Figure 5:**
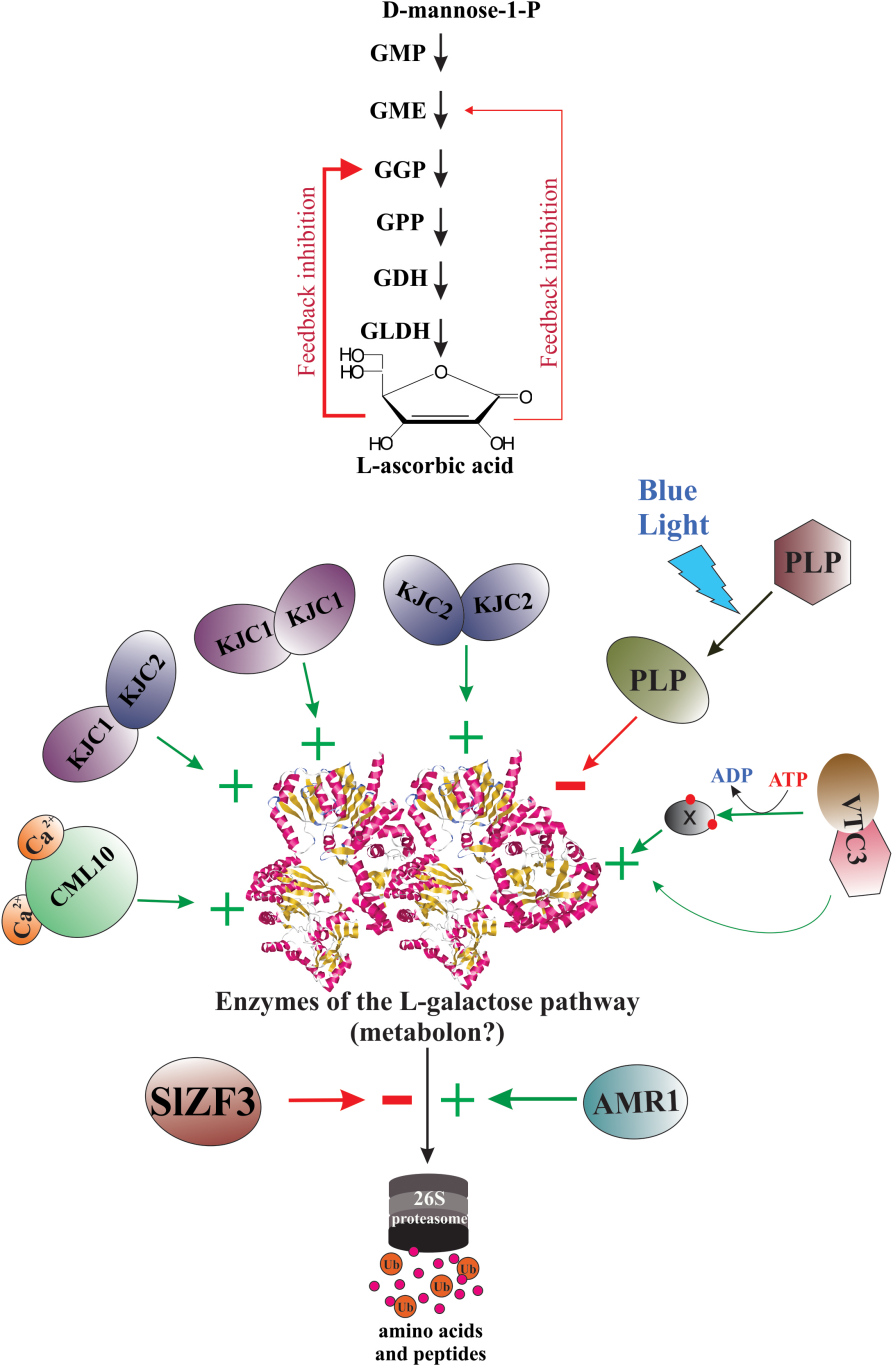
Biochemical strategies for regulation of AsA biosynthesis in plants. In plants, several biochemical mechanisms have been described, including control of the enzyme levels, control of the enzyme catalytic activity, feedback inhibition of regulatory enzymes, and other processes.

The molecular and biochemical fundaments of the regulatory mechanism of AsA content in plant tissues by controlling enzyme levels have been partially unraveled. In this context, investigations on thale cress found that protein levels of the GMP enzyme correlate with changes in AsA contents under illumination and darkness conditions. Thus, under illumination, the protein level of GMP and AsA content increases, and a contrasting result occurs under darkness. The significant decrease in GMP protein levels under darkness is attributed to ubiquitination-dependent enzyme degradation through the 26S proteasome pathway (**Figure 5**). It was experimentally verified that the photomorphogenic factor COP9 signalosome subunit 5B (CSN5B) interacts with the amino-terminal end (the first 40 amino acid residues) of the enzyme and then associates with the COP9 signalosome complex (Wang et al., 2013). Furthermore, these researchers found that an aspartic acid residue at position 27 of the amino-terminal end of the GMP enzyme is the key-binding site to interact with CSN5B. Consequently, a mutation in this position (i.e., D27E) impairs the interaction with CSN5B and improves the stability of the GMP enzyme, thereafter boosting the biosynthesis and accumulation of AsA (Li et al., 2016). These researchers also indicate that the mutation D27E in the GMP enzyme of thale cress is coincident with this site in other plant species. These plant species include *Citrus clementina* “clementine”, *Citrus sinensis* “sweet orange”, *Solanum lycopersicum* “tomato”, and *Malpighia glabra* “acerola”. Consequently, the plant species containing the mutation D27E in the GMP enzyme could produce higher AsA contents than other plant species that lack this mutation (Li et al., 2016)

The first discovery of a protein acting as a negative regulator of gene-encoding enzymes of the L-galactose pathway for AsA biosynthesis was reported by Nessler’s research team in the late 2000s (Zhang et al., 2009). These researchers observed that when the transcripts of the gene *At1g65770*, named *AMR1* (ascorbic acid mannose pathway regulator 1), accumulate, the AsA content decreases in cells of thale cress, which is associated with the reduced expression level of the genes encoding the six enzymes of the L-galactose pathway for AsA biosynthesis (GMP, GGP, GPP, GME, GDH, and GLDH). Both factors, the plant development stage and the light intensity, control the expression levels of the *AMR1* gene. Thus in one-week-old plants, AsA content is high (5.4 μM.g^−1^ fresh weight) and gradually decreases with age until 1.5 μM.g^−1^ fresh weight in five-week-old plants. Simultaneously with AsA content decrease, the gene expression level of *AMR1* increases over time, showing the lowest value at one-week-old plants and reaching the highest value in five-week-old plants. *AMR1* transcripts increase under low-light conditions (≈50 μE.m^−2^. S^−1^) at different developmental stages of the plants. In contrast, under high-light conditions (≈200 μE.m^−2^. S^−1^), the gene expression level of *AMR1* decreases significantly at all plant ages. The AMR1 protein contains an F-box domain at its amino-terminal region (amino acid residues from 4 to 50) (Zhang et al., 2009). The F-box domain is a receptor for ubiquitination targets, and proteins with these domains are involved in targeted proteolysis and are known to form SCF (for Skp1-Cullin-F-box)-ubiquitin-E3 ligase complex, which recognizes both the E2 protein, having activated ubiquitin and the protein substrate targeted for ubiquitination (Moon et al., 2004; Xu and Xue, 2019; Kandolf et al., 2022). Although the researchers did not identify the protein target(s) for AMR1 for a better comprehension of the molecular mechanism of AsA regulation and control of the gene expression, AMR1 appears to play a key role in modulating AsA levels in thale cress through the negative regulation of genes encoding enzymes of the main biosynthetic pathway for AsA in response to developmental and environmental signals (Zhang et al., 2009). Furthermore, a Chinese research team found that both the mRNA and the protein levels of AMR1 Like 1 of *Malus domestica* “apple” (AMR1L1), negatively correlate with AsA contents during the development of apple fruit. Also, in the leaves of AMR1L1-overexpressing apple lines, the mRNA levels of the *GMP* gene are highest because the transcription factor ERF98 binds to its promoter to boost the transcription. However, the corresponding translated protein level and the enzyme’s catalytic activity are significantly lowest because the AMR1L1 protein binds to the GMP enzyme and promotes its hydrolysis *via* ubiquitination-dependent degradation through the 26S proteasome pathway, thus controlling AsA biosynthesis at the post-translational level (Ma et al., 2022).

Additionally, a novel regulatory gene that affects the GMP enzyme levels was identified recently in *Solanum lycopersicum* “tomato” and thale cress. This regulatory gene, named *SlZF3*, encodes a Cys2/His2‐type zinc‐finger protein, which contains an EAR repression domain. When plants are cultured under salt-stress conditions, the *SlZF3* gene expression is rapidly induced. Then the translated encoded SlZF3 protein forms a complex with CSN5B, depleting its free form, thereby avoiding the complex formation between CSN5B and GMP. Consequently, the SlZF3 protein prevents the hydrolytic degradation of the GMP enzyme *via* the 26S proteasome pathway. Due to this action mechanism, the SlZF3 protein promotes the biosynthesis and accumulation of AsA, improving plant tolerance to this abiotic stress condition (Li et al., 2018). It would be more interesting to test how this molecular system of AsA content regulation, through the synthesis and degradation of the GMP enzyme, works in plants simultaneously exposed to lighting/darkness and salt-stress conditions.

### 5.2 Control of the enzyme catalytic activity

Previously has been proved in thale cress that the PMM enzyme interacts specifically with the calcium sensor named calmodulin-like protein 10 (CML10) (Cho et al., 2016). The protein complex formed by the PMM enzyme and CML10 (with an interaction ratio of 1:2) occurs in the cytoplasm in the presence of Ca^2+^. The researchers also demonstrated that under *in vitro* assays, the interaction significantly augments the catalytic activity of the PMM enzyme. Thus, without CML10, the *V*_max_ of the PMM enzyme is 1.166 μmol.mg of protein^-1^.min^−1^, but when the reaction mix contains 5.0 μg of CML10, the *V*_max_ of the PMM enzyme increases to 1.492 μmol.mg of protein^-1^.min^−1^ (*V*_max_ increase ≈28%). In addition, the authors showed that under an oxidative stress condition (H_2_O_2_-treated wild-type plants), *CML10* mRNA levels and AsA levels increase rapidly and significantly, but the expression level of the *PMM* gene does not change. These results suggest that when the plants are subject to oxidative stress, the expression of the *CML10* gene is induced in parallel with intracellular Ca^2+^ increase. Subsequently, the synthesized CML10 protein binds calcium ions, suffers a conformational change, and complexes with the PMM enzyme. Thus, boosting the biosynthesis of AsA and enabling the plants to overcome oxidative stress (Cho et al., 2016).

A novel biochemical mechanism to activate the catalytic activity of the GMP enzyme was recently discovered by Japanese researchers. The research team identified in thale cress two nucleotide sugar pyrophosphorylase-like proteins, KONJAC1 (KJC1) and KJC2. These proteins form homo- and heterodimeric complexes with the GMP enzyme. The formation of these protein complexes significantly stimulates the catalytic activity of the GMP enzyme. The researchers show that *in vivo* assays, the lack of KJC1 decreases the catalytic activity of the GMP enzyme until 90%. However, *in vitro* assays using a recombinant version of KJC1, the researchers proved that this protein boosts the activity of the GMP enzyme only 2-fold. These significant differences could be attributed to the nature of the *in vitro* and the *in vivo* conditions. This could be due to the stabilizing role of KJC proteins in the GMP enzyme (Sawake et al., 2015).

Also, it was observed that the catalytic activity of the GME enzyme of thale cress could be controlled by interaction with heat-shock proteins. During the purification of both native and recombinant GME enzymes was reported that these enzymes co-purify with the Hsc70.3 protein (heat-shock cognate 70.3) of *Arabidopsis thaliana* and the DnaK (the prokaryotic analog of hsp70, the eukaryotic 70 kDa heat-shock protein) of *Escherichia coli*, respectively (Wolucka and Van Montagu, 2003). When the majority of the Hsc70.3 protein was separated from the GME enzyme by gel filtration, the catalytic activity of the GME enzyme was reduced ten-fold. This significant reduction of the catalytic activity could be attributable to the disruption of the formed complex between the GME enzyme and the Hsc70.3 chaperone. Based on these results, the researchers hypothesized that the Hsc70.3 protein might be implicated in the folding and/or regulation of the GME enzyme activity (Wolucka and Van Montagu, 2003).

In addition, it was demonstrated that the enzyme responsible for producing AsA from GL has a critical cysteine residue (C340) in the substrate binding site that suffers reversible oxidation (redox sensitive-thiol). Oxidation of this cysteine residue by hydrogen peroxide inactivates the GLDH enzyme (Leferink et al., 2009a). Consequently, the catalytic activity of the last enzyme of the L-galactose pathway for AsA biosynthesis is susceptible to oxidative stress. Suggesting that the GLDH enzyme could be regulated, at least in leaves, by reversible oxidative modification induced by light/dark cycles. The molecular mechanisms supporting the light/dark and photosynthesis-dependent regulation of AsA biosynthesis in photosynthetic tissues/organs stay incomprehensible.

Another possible player in the regulation of AsA biosynthesis at the post-translational level is the *At2g40860* gene (*VTC3* gene) located on *Arabidopsis thaliana* chromosome 2. This gene has been identified by positional cloning (Conklin et al., 2013) using the AsA-deficient *Arabidopsis* vitamin C (vtc) mutants (*vtc3-1* and *vtc3-2*) (Conklin et al., 2000). The gene is required to respond to high temperature/continuous light stress through the upregulation of AsA in the plant cells (Conklin et al., 2013). Although the *VTC3* gene was predicted to encode a bifunctional chloroplastic protein composed of an amino-terminal catalytic domain of a protein kinase and a carboxy-terminal Ser/Thr protein phosphatase 2C domain (Conklin et al., 2013) and thale cress plants with mutations in the *VTC3* gene (*vtc3-1* [G202E missense mutation] and *vtc3-2* [Q448X nonsense mutation]), which modifies the open reading frames in both domains, are lower AsA producers (Conklin et al., 2000; Conklin et al., 2013). To date, however, it was not tested whether the VTC3 protein functions as a protein kinase and/or phosphatase. With the possession of two signal transduction domains (protein kinase and protein phosphatase), and depending on the environmental stimulus, the VTC3 protein could phosphorylate or dephosphorylate another protein target. Alternatively, the bifunctional protein could be autophosphorylated or autodephosphorylated. In either situation, it was hypothesized that the VTC3 protein could modify the catalytic activity of an AsA biosynthetic enzyme(s) through a signal transduction cascade that transduces an environmental stimulus to increase the AsA pool size (Conklin et al., 2013). According to this hypothesis, a phosphoproteomic study of thale cress has shown that the PMM, GME, and GGP enzymes are phosphorylated (Mergner et al., 2020). However, it is unknown if phosphorylation influences the catalytic activity of these enzymes. Consequently, it is pending to demonstrate if the VTC3 protein possesses kinase and/or phosphatase activity and how are their molecular mechanisms responsible for AsA biosynthesis regulation in the plant cells.

Recently also was discovered a novel biochemical mechanism that regulates the catalytic activity of the GGP enzyme of *Solanum lycopersicum*. The protein involved in this unique regulatory mechanism is encoded by the *Solyc05g07020* gene. The protein belongs to a new class of photoreceptor proteins containing a LOV (Light, Oxygen, and Voltage) domain, which is named PAS/LOV protein (PLP). The PLP protein interacts with the GGP enzyme in the cytoplasm and the nucleus. In this interaction, the PLP protein acts as a non-competitive inhibitor of the GGP enzyme, thus significantly decreasing its catalytic activity. Interestingly, the formation of the PLP protein-GGP enzyme complex is modulable by blue light exposure. Because blue light inactivates the formation of these inhibitory complexes by inducing conformational changes in the PLP protein. Consequently, when plants are exposed to blue light, the inhibitory effect of PLP is avoided, and the catalytic activity of the GGP enzyme is boosted. Thus, the AsA content in leaves is increased significantly (https://www.biorxiv.org/content/10.1101/2022.12.12.520143v1).

### 5.3 Feedback inhibition of regulatory enzymes

Some investigations provide experimental evidence that the *de novo* biosynthesis of AsA could be regulated by feedback inhibition. The first study conducted by (De Gara et al., 1989) demonstrated that the rate of AsA biosynthesis in tubers of *Solanum tuberosum* correlates with the AsA content in the cells. Thus, when the intracellular AsA content is at the lowest level, its biosynthesis reaches a maximum. Also, when the intracellular AsA content is highest, the rate of AsA biosynthesis is virtually zero. Under the last experimental condition, when the researchers added the first precursor of the metabolic pathway (α-D-glucose), it did not induce AsA biosynthesis. However, the addition of L-galactono-1,4-lactone (the last precursor) induces a high rate of AsA biosynthesis (De Gara et al., 1989). These results suggest that *in vivo* AsA biosynthesis is subject to a regulatory mechanism that controls an initial step in the biosynthetic pathway. The last catalytic reaction of the biosynthetic pathway, which is catalyzed by the GLDH enzyme, is never inhibited and, moreover, its catalytic activity is greater than that of the preceding reactions catalyzed by other enzymes (De Gara et al., 1989).

The second investigation aimed to establish if AsA biosynthesis and turnover are influenced by the endogenous AsA content in embryonic axes from *Pisum sativum*. The researchers used ^14^C-labelled α-D-glucose to estimate the rate of AsA synthesis and turnover (Pallanca and Smirnoff, 2000). The results show that AsA biosynthesis is robustly blocked by a high AsA content, while the turnover rate is faster and directly proportional to AsA content. The researchers also show that the rate of AsA biosynthesis drops as a linear function of the AsA content, regardless of the exposition time to exogenous AsA. As inhibition of AsA biosynthesis occurs in the first three hours of AsA feeding, this inhibitory effect could be caused by feedback inhibition of AsA biosynthetic enzymes. However, the researchers suggest conducting more experiments to differentiate between feedback inhibition and repression of the genetic expression of genes encoding enzymes of the biosynthetic pathway (Pallanca and Smirnoff, 2000).

The third study demonstrated that AsA at the physiological concentration (1 mM) inhibits the catalytic activity of the GME enzyme by 15%. This decrease in enzyme activity suggests a feedback inhibition of the GME enzyme by AsA, the final product of the L-galactose pathway (Wolucka and Van Montagu, 2003). Furthermore, to determine if the regulation of AsA biosynthesis by feedback inhibition occurs *in vivo*, the researchers conducted *in vivo* labeling experiments using a cellular suspension of thale cress treated with D-[U-^14^C]-mannose. The results show that when the cellular suspension was fed with AsA, the intracellular level of AsA increased, but the incorporation of radioactive D-[U-^14^C]-mannose into AsA decreased, so the feedback inhibition of AsA biosynthesis by AsA was clearly observed *in vivo* (Wolucka and Van Montagu, 2003).

Also, a Japanese research team suggested that AsA acts as a feedback inhibitor of the recombinant GDH enzyme from *Spinacia oleracea* “spinach” (Mieda et al., 2004). However, a recent publication shows that the recombinant GDH enzymes both from *Myrciaria dubia* “camu-camu” and *Spinacia oleracea* “spinach” are refractory to inhibition by AsA (Vargas et al., 2022). Thus, the decreased activity of the GME enzyme in increased concentrations of AsA, previously reported by (Mieda et al., 2004), could be related to a decrease in the pH values of the reaction medium (Vargas et al., 2022).

Recently was constructed a kinetic model based on the properties of enzymes of the L-galactose pathway for AsA biosynthesis (Fenech et al., 2021). The model includes inhibition of the GGP enzyme by AsA and the proposed translational repression *via* the 5’-uORF. The kinetic model displays that the only enzymatic reaction in the L-galactose pathway that controls the intermediary metabolites flux and AsA content is catalyzed by the GGP enzyme if the feedback inhibition is sufficiently strong. Decreasing the strength of feedback inhibition by AsA on the GGP enzyme, at some point, decreases the intermediary metabolites flux and the concentration control coefficients. The kinetic model predicts that the enzymatic reactions after the GGP enzyme exert little control and that in the presence of feedback inhibition, the enzymatic reaction catalyzed by the GGP enzyme is the unique critical step (Fenech et al., 2021). However, it is necessary to conduct novel experiments to verify these predictions and identify the feedback inhibitors and their target enzymes of the L-galactose pathway for AsA biosynthesis.

### 5.4 Subcellular compartmentation of AsA

AsA has a compartment-specific distribution in the plant cells. Using immunogold labeling, researchers determined that the subcellular compartmentation of AsA shows differences. Thus, in cells of *Arabidopsis thaliana*, the highest AsA concentration is registered in the nucleus (16.3 mM), cytosol (21.7 mM), and peroxisomes (22.8 mM), while chloroplasts (10.8 mM), mitochondria (10.4 mM), and vacuoles (2.3 mM) are the organelles with the lowest AsA contents (Zechmann et al., 2011). As the last enzymatic reaction for the AsA biosynthesis occurs in the mitochondria (Mapson et al., 1954; Bartoli et al., 2000), and neither AsA nor its oxidized forms (i.e., dehydroascorbate and monodehydroascorbate) can diffuse through the phospholipid bilayers (Fernie and Tóth, 2015) the differential distribution of AsA contents among organelles indicates that AsA must be transported from the mitochondria through the cytosol into the chloroplast (Miyaji et al., 2015) and other organelles using specific transport systems (Horemans et al., 2000; Foyer, 2015). The differential distribution of AsA among organelles is fundamental to making pleiotropic functions in plants. These functions of AsA include detoxification of reactive oxygen species, redox signalling, and modulation of gene expression. Also, AsA is very important for the regulation of enzymatic activities. Consequently, the molecular process that controls the complex transport system to have a differential intracellular distribution of AsA is indirectly involved in the regulation of AsA biosynthesis by modifying the degree of feedback inhibition.

### 5.5 Metabolon assembly of the L-galactose pathway enzymes

Recent research suggests that the enzymes involved in AsA biosynthesis through the L-galactose pathway constitute a metabolon (Fenech et al., 2021). This class of enzymatic assembly is fundamental to making the flux of the intermediary metabolites of a specific metabolic pathway more efficient and facilitates their regulation. However, there is still much research to be done to elucidate how this complex protein machinery works and visualize their structures and interactions in a natural context inside the plant cells. An innovative technique to solve problems such as the protein complex cannot be purified intact, or the function of the protein complex is lost outer the cells is the *in situ* observation of these protein complexes using the cryo-electron tomography (Oikonomou and Jensen, 2017; Khanna and Villa, 2022; Saibil, 2022). With these novel technical approaches, researchers will have the ability to identify the heterogenous protein communities that could constitute the metabolon involved in AsA metabolism in plant cells.

### 5.6 Control of AsA biosynthesis by electron flux

Some investigations have demonstrated that photosynthetic electron transport (PET) regulates AsA content in leaves. Thus, the role of photosynthesis in AsA biosynthesis is more than providing the hexose biosynthetic precursors. For example, the supply of GL to leaves of thale cress increases the AsA content in these tissues, dependent on illumination. But treatment with PET inhibitors and GL supply significantly reduces the increases in AsA content under illumination. Thus, light, specifically the redox state of PET, appeared to play a key function in regulating the conversion of GL into AsA in the mitochondria, reflecting the cellular level of AsA in plants (Yabuta et al., 2008). However, the signalling pathway and the mechanisms that control the regulation of AsA content through these processes need to be discovered (Yabuta et al., 2007).

Additionally, it was demonstrated that exists a bidirectional tight regulation between the mitochondrial electron transfer chain (mETC) and the AsA biosynthesis in plants. First, because the last enzyme (GLDH enzyme) of the L-galactose pathway is a component of the mitochondrial complex I, thus its catalytic activity could be regulated by the electron flow across this protein complex. Second, the GLDH enzyme uses oxidized cytochrome c as the only electron acceptor (Millar et al., 2003).

Based on the results of these investigations, it was proposed that AsA can be a key player in the regulatory network that includes the PET, the mETC, and the Krebs cycle. Because the AsA content can influence the intensity of the photosynthesis (Nunes-Nesi et al., 2005; Talla et al., 2011). Also, the electron flux through the PET influences the AsA biosynthesis (Yabuta et al., 2007; Yabuta et al., 2008). In addition, AsA biosynthesis is strongly coupled to the mETC by providing electrons to complex IV when the Krebs cycle is inhibited (Bartoli et al., 2000; Nunes-Nesi et al., 2005). Finally, the activity of alternative oxidase influences the rate of the AsA biosynthesis (Bartoli et al., 2006; Mazorra Morales et al., 2022). Consequently, any change in either of these network players influences the regulatory network (Szarka et al., 2013).

## 6 Conclusions and future directions

We know that the AsA content in plant tissues results from a dynamic equilibrium controlled by complex and little-comprehended regulatory mechanisms. These regulatory mechanisms orchestrates responses to biotic and abiotic environmental cues by enabling or disabling AsA anabolism, catabolism, recycling, and its intra- and intercellular transport and distribution. But until now, our knowledge of plants’ genetic and biochemical strategies to regulate, specifically AsA biosynthesis, is scarce, dispersed, and fragmentary. Thus, in the near future, to gain a deeper understanding of these regulatory processes, it is necessary to use multi-omic approaches together with novel approaches to solving the structural and functional (kinetic parameters) information of the enzymes (individually and protein complexes) and regulatory proteins involved. With the basic information generated and modern tools of genome edition and synthetic biology approaches, we could genetically improve promissory plants to be AsA hyper producers and support animal and human nutrition.

## Author contributions

JC, CG, and MC conceived and designed the manuscript content. JC wrote the manuscript, and CG and MC edited the manuscript. All authors contributed to the article and approved the submitted version.
